# Antibiotic resistance genes in gut of breast-fed neonates born by caesarean section originate from breast milk and hospital ward air

**DOI:** 10.1186/s12866-022-02447-8

**Published:** 2022-01-29

**Authors:** Kunming Zhang, Min Jin, Dong Yang, Zhiqiang Shen, Weili Liu, Jing Yin, Zhongwei Yang, Huaran Wang, Danyang Shi, Jiping Yang, Haibei Li, Yaqiong Chen, Zhixian Gao, Zhigang Qiu, Haixia Shi, Jun-Wen Li

**Affiliations:** 1 Department of Environment and Health, Tianjin Institute of Environmental and Operational Medicine, Key Laboratory of Risk Assessment and Control for Environment & Food Safety, No. 1 Dali Road, Tianjin, 300050 China; 2 Characteristic Medical Centre of the Chinese People’s Armed Police Force, No. 220 Chenglin Road, Tianjin, 300162 China

**Keywords:** Antibiotic resistance genes, Gut, Newborns, Originate, Breast Milk and hospital ward air

## Abstract

The human gut is a reservoir of antibiotic resistance genes (ARGs). Even in the absence of antibiotics, ARGs are present in large quantities in faeces of adults, children and even newborns. However, where and when ARGs are acquired remains unclear, as does the types of ARGs acquired. Herein, we recruited 82 pairs of women and their caesarean section newborns. Conventional culture methods and quantitative PCR were employed to detect nine species and six ARG types in meconia, faeces from 3-day-old newborns, amniotic fluid, colostrum, and hospital ward air samples. Furthermore, ARG transfer was explored by tracking *Staphylococcus epidermidis* isolated from faeces of 3-day-old newborns, colostrum and ward air samples using multi-locus sequence typing (MLST). No ARGs or microorganisms were detected in meconia or amniotic fluid. One or more ARGs were detected in 90.2% of faeces from 3-day-old newborns, and the *mecA* gene exhibited the highest detection rate (45.1%). ARGs were detected in 85.4% of colostra consistent with ARGs in faeces from 3-day-old newborns. Some ARGs were detected in ward air, and might also be a source of ARGs in neonatal faeces. Isolation of *S. epidermidis* from neonatal faeces was consistent with antibiotic resistance and gene profiles for colostrum samples. Traceability analysis of *S. epidermidis* showed that ARGs in neonatal faeces mainly originated from colostrum, and partly from ward air. After birth, neonates born by caesarean section obtain a variety of ARGs mainly from colostrum, and partly from ward air.

**Supplementary Information:**

The online version contains supplementary material available at 10.1186/s12866-022-02447-8.

## Introduction

The gut is the largest digestive organ of the human body, houses a complex microbial flora and acts as a reservoir of antibiotic resistance genes (ARGs) [1]. Worldwide, more than 1000 species and > 50 types of ARGs have been detected in hundreds of adult intestine samples [2, 3]. In addition to the adult gut, antibiotic-resistant bacteria and ARGs have also been detected in large quantities in children’s faeces, and even in neonatal meconia [1, 4–6]. However, it is not clear when ARGs appear in the gut of newborns. It is reported that ARGs were found the earliest in neonatal faeces 1 week after birth or later meconia [1, 4–6].

ARGs in human gut may be acquired from antibiotic-resistant bacteria in the uterus, during delivery through the vagina or after birth. Previous reports suggested that the amniotic fluid and/or the placenta contain a variety of bacteria that seed the neonatal intestinal flora [7–9], but Marcus C. de Goffau et al. recently concluded that human placenta did not have a microbiome [10]. The birth canal contains a large number of bacteria that may include ARGs, and during childbirth neonates may acquire bacteria from the vagina [11]. Antibiotic-resistant bacteria may also be transmitted to newborns after birth through exposure to ward air, drinking water and breast milk, since ARGs have been detected in these media [12–18]. However, previous studies do not exclude the effects of bacteria in the birth canal during delivery and the use of antibiotics and probiotics by mothers when they concluded that bacteria in the mother’s gut and breast milk might be a source of ARGs in the gut of newborns [19]. Additionally, several studies included too few subjects to achieve statistically significant conclusions [19, 20]. Therefore, these studies could not directly confirm the source of ARGs. Indeed, where and when ARGs are acquired during childbirth remains unclear, as does the types of ARGs acquired. It is possible that the child has acquired drug-resistant bacteria after birth. Once the infant’s immunity is low and attacked by drug-resistant pathogenic bacteria, it will lead to health problems that difficult to treat, such as pneumonia, sepsis and diarrhea,. By finding out “where and when ARGs are acquired during childbirth”, we hope to take relevant measures to eliminate the contact of neonatal drug-resistant bacteria and reduce or prevent the threat of drug-resistant bacteria to newborns.

In the present study, 82 pairs of women and their caesarean section newborns were assessed to identify the initial sources of ARGs in the faeces of newborns. Colony culture and quantitative PCR methods were applied to detect bacteria and determine the type and abundance of ARGs in the amniotic fluid and colostrum of the mother, the first meconium and the first normal faeces on the third day of newborn, hospital ward air and drinking water. In order to elucidate when and from where ARGs are acquired by newborns, ARG transfer was explored by tracking *Staphylococcus epidermidis* isolates from neonatal faeces, colostrum and air samples using multi-locus sequence typing (MLST).

## Materials and methods

### Subjects

In total, 82 pairs of mothers and newborns were recruited from the obstetrics department of the Affiliated Hospital of Logistics University of People’s Armed Police Force, Tianjin, China. Mothers were 29.30 ± 0.44 years old. The inclusion criteria: mothers had no diabetes, hypertension or infectious disease cases during pregnancy, and no history of antibiotics or probiotics during the last 6 months before delivery; and all neonates were born by caesarean section through full-term delivery, were of normal weight, had no congenital diseases, and no history of antibiotic use (48 males and 34 females were included); all the infant were exclusively breastfed and strictly nurseries in babies room in the hospital. This study was approved by the ethical committees of the Affiliated Hospital of Logistics University of People’s Armed Police Force and the Institute of Environmental and Operational Medicine. Our research has been performed in accordance with the *Declaration of Helsinki*. Pregnant woman and her family members were informed of sample collection and risks, and all appropriate consent forms were signed before initiation of the study.

### Sample collection

Doctors responsible for collecting samples were trained in advance to ensure that samples were not contaminated. For neonatal faeces collection, meconium and the first normal faeces was collected within 3 h after birth, and on the third day after birth (48–72 h). After newborns had defecated onto paper diapers, ~ 2 g samples from the middle part of faeces were collected, avoiding contact with paper diapers, placed in faecal collection tubes on ice, and immediately delivered to the laboratory. For amniotic fluid collection from mothers, during caesarean sections, professional physicians extracted 10 ml of amniotic fluid under aseptic operation conditions, placed samples on ice, and samples were immediately delivered to the laboratory. For colostrum collection, nipples were wiped with a soapy towel before sampling, the first 2 drops of milk were discarded, and ~ 2 ml colostrum samples were collected, placed on ice, and immediately delivered to the laboratory. For air microbial collection, three wards were chosen at a time, and 12 times in total on different days. In order to exclude the bacteria on the skin into breast milk, the nipple and surrounding skin were cleaned twice with normal saline cotton swab before breast-feeding. There were 10 wards and four newborns in each ward, which was cleaned by ventilation twice a day. According to the traditional passive sedimentation method of the Hospital Sanitary Disinfection Standard (GB15982–2012) [21], three brain heart infusion (BHI)-agar plates (BD, New Jersey, USA) and a blank sample were placed in each ward at 8–8:30 am (before indoor disinfection). The plates were immediately delivered to the laboratory in cold storage, and incubated at 37 °C for 24 h in aerobic conditions. Air samples were collected once a week throughout the collecting period of other samples, a total of 12 samples were collected. After incubation, the microorganisms were gathered as an air sample by washing the surface of the plate with sterile water, centrifuging and collecting the sediment”. Therefore, air collection does not involve the collected volume. For water collection, first disinfected the faucet with 75% alcohol, then drained the water for 5 min, then collected 500 ml with sterilized bottle, and water samples were immediately transported to the laboratory in cold storage and were processed within 4 h. The total microorganisms in triplicates were collected by filtration using a 0.45-μm cellulose ester membrane (Millipore Corp., USA), and then plated on brain heart infusion (BHI)-agar plates, which were incubated at 37 °C for 24 h in aerobic conditions. After incubation, the microorganisms were gathered by using the same process as air samples. Bacterial isolation and ARGs detection were prepared according to a previously described method [16].

#### Bacterial isolation and culturing

BHI agar plates were used to recover the total cultivable microbial population of colostrum, faeces or meconium in anaerobic conditions at 37 °C for 48 h. Each sample was repeated in triplicate.

Isolation and culturing of *S. epidermidis* were performed as follows: colostrum and faeces samples were diluted with 0.9% saline, amniotic fluid samples were not diluted, and 100 μl were coated directly onto Baird-Parker agar (BD) for *Staphylococcus* selection and incubated at 37 °C for 24 h. The medium was equilibrated in the ward for 20 min, then incubated at 37 °C for 24 h in aerobic conditions. A single black colony surrounded by a transparent lecithin ring was picked and inoculated into blood agar medium and incubated at 37 °C for 24 h. A single white colony was chosen subsequent experiments. Suspected colonies with inoculation rings were transferred to 30 μl of enzyme-free water, and DNA was obtained by heating at 90 °C for 10 min. Primers used for amplification of *S. epidermidis-*specific fragments were J-StGen (5′-TGGCCAAAAGAGACTATTATGA-3′; forward primer) and J-StEpi (5′-CCACCAAAGCCTTGACTT-3′; reverse primer) [22]. The target fragment confirming the presence of *S. epidermidis* was 249 bp.

#### DNA extraction

A Stool DNA Extraction Kit (Tiangen, Beijing, China) was used to extract DNA from 200 mg faeces and amniotic fluid following the kit instructions. A 10 ml sample of amniotic fluid was centrifuged at 10,000×*g* for 10 min, the supernatant was removed, and the pellet was used for extraction of bacterial DNA. Because the amount of bacterial in air, drinking water and colostrum samples may be very low, there may be fewer drug-resistant genes involved and can not be detected. Considering that the intestinal tract of newborns may also be a bacteria amplification environment, even a small amount of bacteria or drug-resistant genes may reproduce in large quantities. Therefore, air, drinking water and colostrum samples were cultivated and parallel blank samples run during the whole process. Further, in order to avoid contamination, the experiments were performed in the second-level biosafety laboratory and strictly followed the procedure of aseptic operation.

Colostrum, air and drinking water samples were cultured for 48 h and a Bacterial DNA Extraction Kit (Tiangen, Beijing, China) was used to extract DNA.

#### Detection of ARGs by conventional PCR and quantitative PCR

Nine ARGs (*tetM*, *mecA*, *blaTEM*, *ampC*, *ermB*, *sul2*, *aac(6)-Ib*, *blaNDM-1-1* and *mcr-1*) were detected by conventional PCR and quantitative PCR (qPCR). The sequences of primers used to detect ARGs and the amplification conditions are listed in Table S1 and S2. qPCR amplification was performed using an Applied Biosystems 7300 Real-Time PCR System (Applied Biosystems, Foster City, CA, USA) using FastStart Universal SYBR Green Master (Rox) (Roche Diagnostics, Basel, Switzerland) according to the manufacturer’s instructions. The standard thermal profile for PCR amplification was 50 °C for 2 min, 95 °C for 10 min, and 40 cycles of 95 °C for 15 s and 60 °C for 60 s.

#### Antibiotic resistance of *Staphylococcus epidermidis*

Referred study and our pre-experiments show that *S. epidermidis* can be isolated from the human intestine tract and colostrum, and drug resistance is high [23]. *S. epidermidis* was selected for traceability of ARGs because of its high abundance, isolation and resistance rate in the samples. *S. epidermidis* from different sources was tested for resistance to 10 antibiotics using the K-B method or by determining the minimal inhibitory concentration according to the standards issued by CLSI in 2012 (https://clsi.org/). The 10 antibiotics were ampicillin, cefotaxime, penicillin, tetracycline, erythromycin, kanamycin, vancomycin, ofloxacin, chloramphenicol and trimethoprim (Sangon Biotech, Shanghai, China).

#### Multi-locus sequence typing (MLST) of *S. epidermidis*

DNA extracted from *S. epidermidis* was used as a template. Seven housekeeping genes of *S. epidermidis* were amplified by PCR and further sequenced to determine the number of each locus [24]. Specific primers were designed for the seven housekeeping genes and are listed in Table S3.

PCR products were verified by agarose gel electrophoresis, target products were sequenced, and sequences were submitted to the *Staphylococcus epidermidis* MLST database (https://pubmlst.org/organisms/staphylococcus-epidermidis), which returned sequence codes. Strains with all seven corresponding gene codes were searched against the database and MLST numbers were returned, giving the sequence type of the strain.

#### Statistical analysis

Chi-square tests were used to investigate the effect of gender on intestinal ARG colonisation in neonates. To explore the relationships of ARGs in colostrum and neonatal faeces, the frequency of each ARG in colostrum samples was compared with that in faeces by McNemar tests and Pearson correction. In addition, the ratio of the number of ARGs detected simultaneously in colostrum and faeces samples relative to that detected in faeces alone was used as the coincidence rate to evaluate the possibility that ARGs in faeces originated from colostrum. SPSS19.0 was used for statistical analysis.

## Results

### ARGs in faeces of neonates

**Fig. 1:**
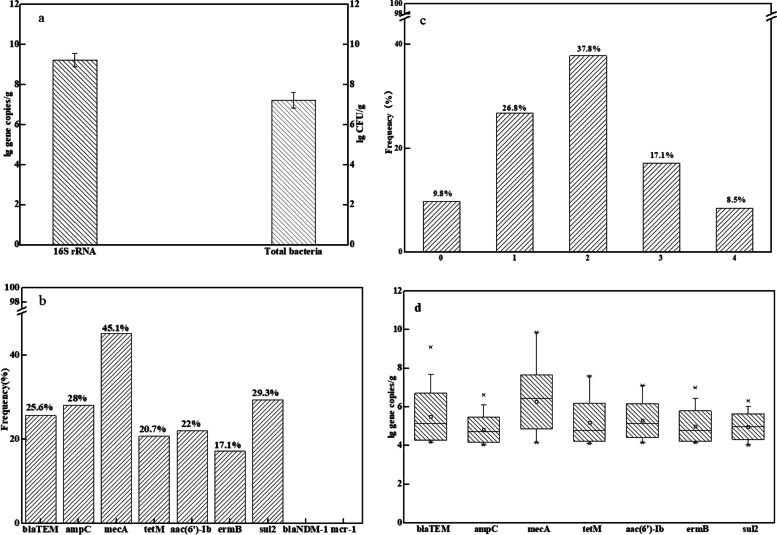
The abundance of bacteria and antibiotic resistance genes (ARGs) in faeces of 3-day-old newborns (FT) (*n* = 82). **a**, Logarithm of the 16S rRNA copy number and colony number per g of FT; **b**, Types and frequencies of ARGs in each FT sample; **c**, Frequencies of each ARGs in FT; **d**, Logarithm of the number of ARGs per g of FT

No bacteria were detected in meconium samples from caesarean section newborns (16S rRNA analysis by quantitative PCR was negative), and no ARGs were detected in meconium samples. In faeces from 3-day-old neonates, the bacterial colony count detected using BHI medium was 10^6^ CFU/g ~ 10^9^ CFU/g. The number of 16S rRNA genes was 10^9.23 ± 0.33GC/g (Fig. 1a).

One or more of the seven ARGs were detected in 90.2% of the first normal faeces. 26.8% contained one of the seven ARGs, 37.8% contained two, 17.1% contained three, and four ARGs were detected in each one sample, accounting for 8.5% (Fig. 1b). The frequency of *blaTEM*, *ampC*, *mecA*, *aac(6)-ib*, *ermB*, *sul2* and *tetM* in faeces of 3-day-old neonates was 25.6, 28, 45.1, 22, 17.1, 29.3 and 20.7%, respectively. The frequency of *mecA* was the highest, but *blaNDM-1* and *mcr-1* were not detected (Fig. 1c). The abundance of ARGs ranged from 7.33 × 10^9^ copies/g to 1.26 × 10^6^ copies/g, among which the *mecA* gene was the highest and *ampC* was the lowest (Fig. 1d). There were no significant differences in the frequency or abundance of ARGs in faeces between male and female newborns (*p > 0.05*; Table S4). We detected ARGs in the faeces of six newborns on 7th and 30th day. The ARGs spectrum were the same as those in the 3rd day. Although the abundance of each ARG fluctuated to some extent (*ermB* and *aac(6′)-Ib* increased, others decreased slightly), it remained stable in general (Fig. S1). It shows that the drug resistance gene is stably colonized in the intestine with microorganisms after entering the intestine of newborns for at least 1 month, indicating that some drug-resistant bacteria in newborns come from the early acquisition of the intestine rather than the use of antibiotics.

### ARGs in colostrum, ward air, drinking water and amniotic fluid samples

**Fig. 2:**
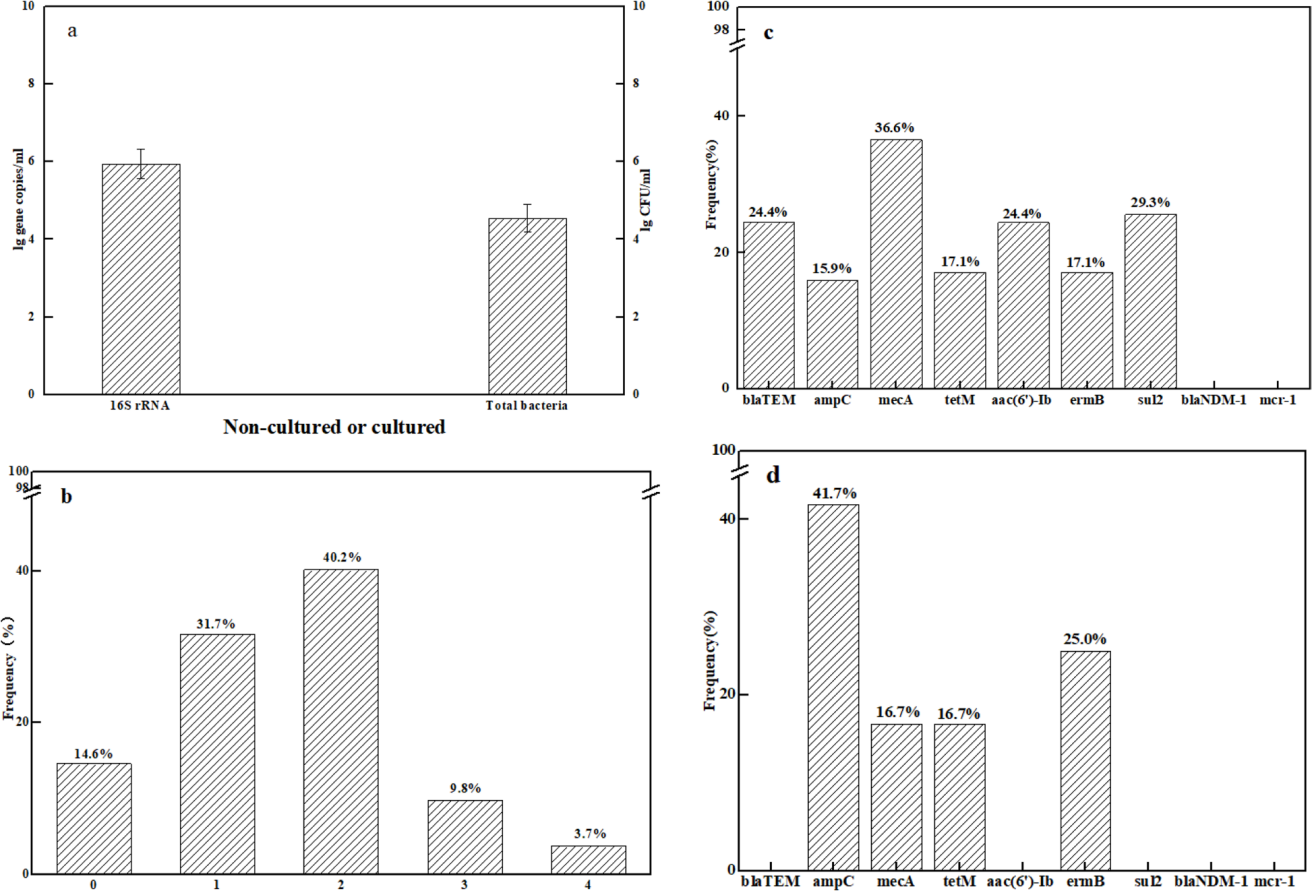
ARGs in colostrum and ward air samples (*n* = 82). **a**, Logarithm of 16S rRNA copy number and colony number in colostrum per ml; **b**, Types and frequencies of ARGs in each colostrum sample; **c**, Frequencies of each ARGs in colostrum samples; **d**, Frequencies of each ARGs in ward air

In colostrum samples, the bacterial colony counts detected using BHI medium were 1.04 × 10^4^ CFU/ml ~ 6.8 × 10^4^ CFU/ml. The copy number of 16S rRNA was 1.4 × 10^5^ GC/ml ~ 8.1 × 10^6^ GC/ml (Fig. 2a).

One or more of the seven ARGs were detected in 85.4% of colostrum samples. 31.7% of colostrum samples contained one ARG, 40.2% contained two, 9.8% contained three, and 3.7% contained four (Fig. 2b). The frequency of *blaTEM*, *ampC*, *mecA*, *aac(6)-ib*, *ermB*, *sul2* and *tetM* was 24.4, 15.9, 36.6, 24.4, 17.1, 25.6 and 17.1%, respectively, but *blaNDM-1*and *mcr-1* were not detected. The frequency of *mecA* was the highest (Fig. 2c). The frequency of ARGs in colostrum was highly consistent with that in faeces of newborns, but slightly lower than in faeces samples.

No cultivable bacteria or ARGs were detected in amniotic fluid samples, and no 16S rRNA was detected by quantitative PCR in amniotic fluid samples. Probably due to the sampling volume (only 500 mL), no culturable bacteria and drug resistance genes were detected in the water samples.

Four types of ARGs (*ampC*, *mecA*, *ermB* and *tetM*) were identified in 12 ward air samples after culturing on BHI medium, of which *ampC* displayed the highest frequency (41.7%), but *blaTEM*, *aac(6)-ib*, *sul2*, *blaNDM-1* and *mcr-1* were not detected (Fig. 2d).

### Association of ARGs in the first normal faeces with colostrum and ward air

**Fig. 3:**
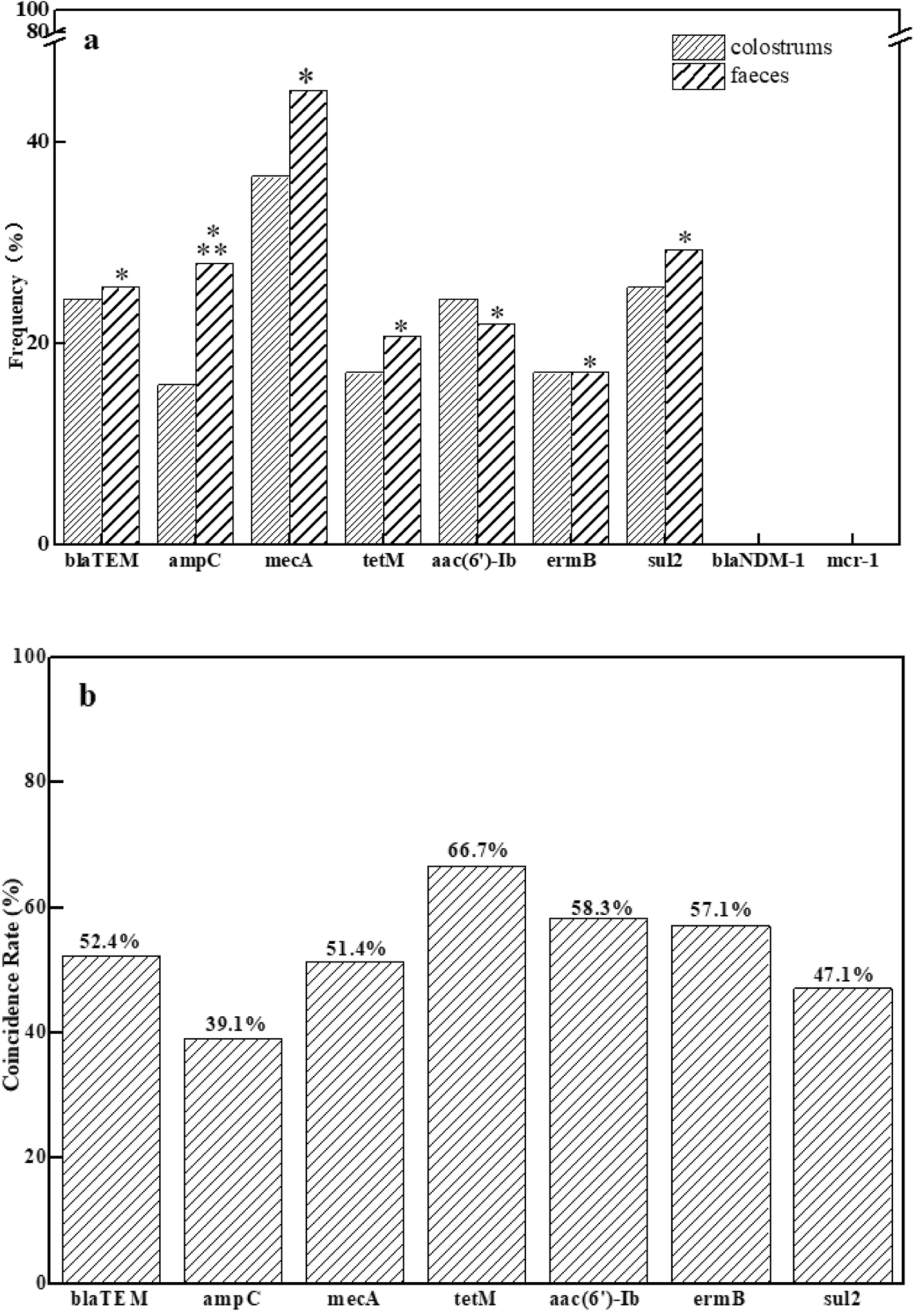
Association of ARGs in the first normal faeces with those in colostrum samples. **a**, Difference and association of the frequencies of ARGs in the first normal faeces and colostrum samples (*n* = 82). *p < 0.05, There is a statistically significant association between the frequency of ARGs in faecal samples and colostrum samples by Pearson correlation test; **p < 0.05, There is a statistically significant difference between the frequency of ARGs in faecal and colostrum samples by McNemar’s chi square test; **b**, Coincidence rates of ARGs in faecal and colostrum samples

McNemar’s chi square tests were used to analyse the relationship between the frequency of ARGs in neonatal faeces and colostrum. Except for *ampC*, there was no significant difference in the frequency of other ARGs between faeces and colostrum samples (*p > 0.05*), indicating that *blaTEM*, *mecA*, *tetM*, *ermB*, *aac(6)-ib* and *sul2* genes were distributed similarly both samples. The frequency of *ampC* in faeces was significantly higher than in colostrum (*p < 0.05*), suggesting that there might be other sources of *ampC* in faeces (Fig. 3a; Table S5).

Pearson correlation analysis was used to determine whether there was a correlation between ARGs in colostrum and faeces. All seven ARGs detected in the first normal faeces showed a positive correlation with those in colostrum (*p < 0.05*), indicating that ARGs in the first normal faeces were mainly derived from the mother’s colostrum (Fig. 3a).

The coincidence rate, the ratio of the number positively detected simultaneously in colostrum and faeces relative to faeces alone, was used to evaluate the possibility that ARGs in faeces originated from colostrum. As shown in Fig. 3b, the probability of *blaTEM*, *ampC*, *mecA*, *tetM*, *aac(6)-ib*, *ermB* and *sul2* in neonatal faeces stemming from colostrum was 52.4, 39.1, 51.4, 66.7, 58.3, 57.1 and 47.1%, respectively. The lowest coincidence rate of *ampC* was observed in colostrum and faecal samples, suggesting that there might be other sources of *ampC* in neonatal faeces.

Meanwhile, four types of ARGs (*ampC*, *mecA*, *ermB* and *tetM*) detected in neonatal faeces were also found in ward air, suggesting that ARGs in neonatal faeces might be partly derived from bacteria in ward air.

### Tracing the origin of ARGs in faeces of neonates

In order to trace the origin of neonatal faecal resistance genes, we analysed the ARG spectra and antibiotic resistance of isolated *S. epidermidis*, and traced the origin using MLST.

**Fig. 4:**
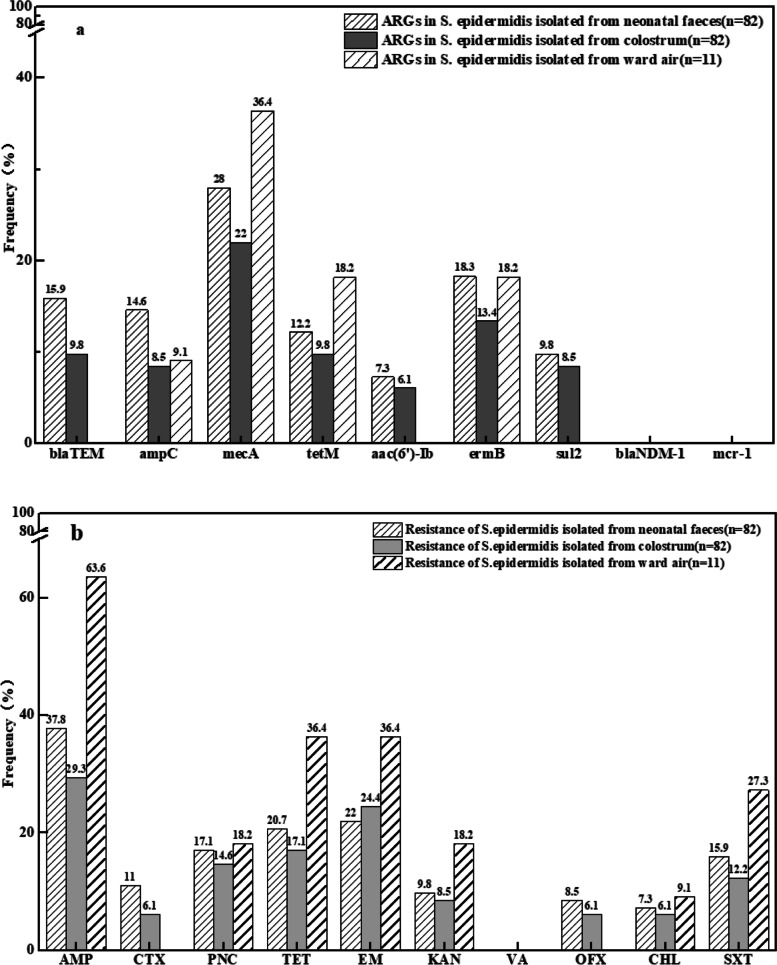
ARGs and resistance of *S. epidermidis* in the first normal faeces, colostrum, and ward air samples. **a**, ARGs in *S. epidermidis* isolated from neonatal faeces, colostrum and ward air, respectively; **b**, Resistance of *S. epidermidis* isolated from neonatal faeces, colostrum and ward air, respectively. AMP = ampicillin. CTX = cefotaxime. PNC = penicillin. TET = tetracycline. EM = erythromycin. KAN = kanamycin. VA = vancomycin. OFX = ofloxacine. CHL = chloramphenicol. SXT = sulfamethoxazole

All seven ARGs (*blaTEM*, *ampC*, *mecA*, *tetM*, *aac(6)-ib*, *ermB* and *sul2*) were detected in *S. epidermidis* isolated from faeces of 3-day-old newborns, and the highest frequency was 28% for *mecA*. These results suggest that the types of ARGs in *S. epidermidis* were similar to those in neonatal faeces (Figs. 1b and 4a). The *S. epidermidis* isolates were resistant to a variety of antibiotics, and resistance was highest for ampicillin (37.8%). The *S. epidermidis* isolates were susceptible to vancomycin (Fig. 4b).

ARGs profiles from *S. epidermidis* isolated from colostrum samples were consistant with those isolated from faeces (Fig. 4a). The antibiotic resistance spectrum of *S. epidermidis* isolated from colostrum samples was consistent with that of *S. epidermidis* isolated from faeces (Fig. 4b).

Four of the seven ARGs (*ampC*, *tetM*, *mecA* and *ermB*) were detected in 11 *S. epidermidis* strains isolated from ward air. The ARGs types were different from those of *S. epidermidis* isolated from faeces and colostrum (Fig. 4a). The antibiotic resistance spectrum was also different from those isolated from faeces and colostrum (Fig. 4b).

### Traceability analysis of *S. epidermidis* by MLST

**Fig. 5:**
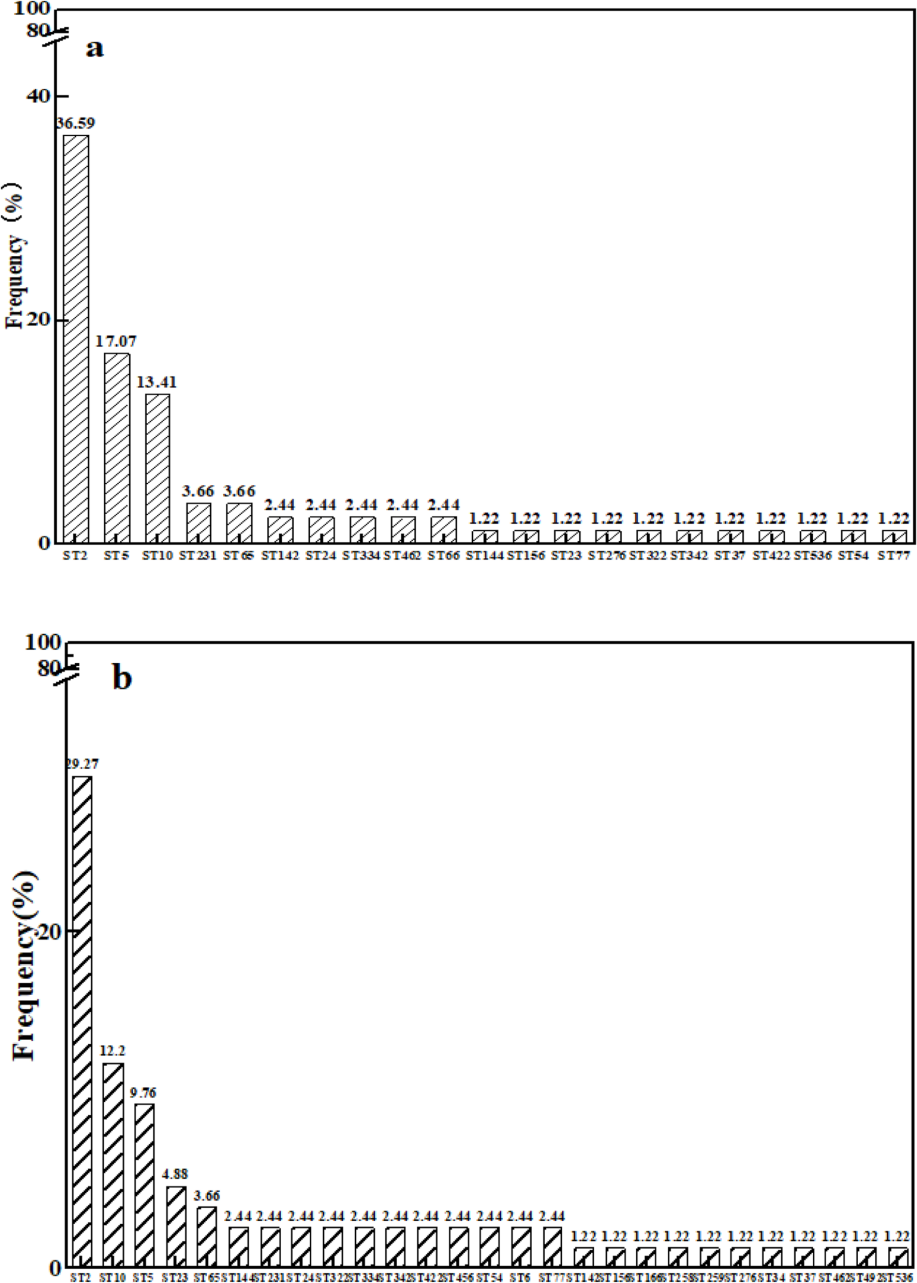
MLST types (ST) of *S. epidermidis* isolated from the first normal faeces and colostrum samples (*n* = 82). **a**, STs of *S. epidermidis* isolated from the first normal faeces; **b**, STs of *S. epidermidis* originating from colostrum

In order to elucidate when and from where ARGs are acquired by newborns, MLST was used to track the ARGs of *Staphylococcus epidermidis* isolates from neonatal faeces, colostrum and air samples. There were 21 MLST types (ST) of *S. epidermidis* isolated from neonatal faeces of newborns. The three most abundant types were ST2 (36.6%), ST5 (17.1%) and ST10 (13.4%; Fig. 5a). There were 27 types of *S. epidermidis* isolated from colostrum samples, of which the three most abundant were ST2 (29.3%), ST10 (12.2%) and ST5 (9.8%; Fig. 5b).

We compared the 82 pairs of *S. epidermidis* isolates from newborn faeces and colostrum. Only the *Staphylococcus epidermidis* with the same of ST number, ARGs and drug resistance phenotype considered similar in terms of the criteria listed. And 52 pairs were consistant in terms of ST, with a homology rate of 63.4%. Therefore, ARGs in neonatal faeces appear to be mainly derived from the mother’s colostrum.

There were 11 strains of *S. epidermidis* isolated from ward air, including five strains of ST2, two strains of ST10, two strains of ST71, one strain of ST6 and one strain of ST59. Additionally, the three air-derived *S. epidermidis* with *tetM*, *mecA*, *ampC* or *ermB* were similar in terms of the criteria listed to the three faeces-derived *S. epidermidis*, meaning the four ARGs are confirmed to be transferred from air to faeces. These results suggest that ARGs in neonatal faeces, particularly *tetM, mecA, ampC* and *ermB*, might also be partially derived from hospital ward air.

## Discussion

In this study, nine types of most common ARGs were detected in 82 pairs of neonatal faeces and maternal amniotic fluid, colostrum samples, ward air and drinking water samples. The nine ARGs are related to resistance to tetracyclines (*tetM*), beta-lactams (*mecA*, *blaTEM*, *blaNDM-1* and *ampC*), macrolides (*ermB*), sulfonamides (*sul2*), aminoglycosides (*aac(6)-ib*) and polymyxins (*mcr-1*). The effects of vaginal bacteria derived from the mother during delivery, maternal illness, and the use of antibiotics and probiotics were excluded in the experimental design and sample collection. One or more ARGs were detected in more than 90.2% of the first normal faeces, and in more than 85% of colostrum samples, consistent with the spectra of ARGs in neonatal faeces. Traceability analysis of *S. epidermidis* isolated from neonatal faeces and colostrum samples showed that *S. epidermidis* and ARGs were mainly derived from colostrum, although some *S. epidermidis* strains and some ARGs might be derived from ward air.

Whether ARGs in the neonatal intestinal tract are acquired in the prenatal foetal period or during or after birth has always been controversial. To date, no ARGs have been detected in placenta or amniotic fluid. In the present study, neither culturable bacteria nor ARGs were detected in the meconium of newborns or amniotic fluid, though it has been reported that bacteria have been detected from meconium [8] Therefore, the conclusion that ARGs in neonatal intestine originate from amniotic fluid and placenta before birth remains to be confirmed.

It is also believed that ARGs in the neonatal intestine may originate from the external environment after childbirth, including colostrum, because ARGs can be detected in these media [12–15]. We detected one or more ARGs in 90.2% of faeces from 3-day-old neonates, with some in high abundance (*mecA* = 7.33 × 10^9^ copies/g). Moreover, the ARGs spectrum and abundance in faeces remained stable within 30 days after birth. Because there were no ARGs in amniotic fluid or meconium samples, and since bacterial contamination of the birth canal can be discounted, we can infer that ARGs in the faeces of newborns were acquired after birth (e.g. via breast milk and/or air or drinking water).

Antibiotic resistance has also been detected in bacteria isolated from breast milk, including *Staphylococcus*, *Streptococcus* and *Enterococcus* resistant to a variety of antibiotics [13]. *S. epidermidis* can be isolated from the human intestine tract and colostrum, and drug resistance is high [22, 25, 26]. *S. epidermidis* isolated from breast milk can carry a variety of ARGs including *mecA* and *ermB* [12]. In addition, many kinds of antibiotic-resistant bacteria and genes have been detected in hospital ward air and drinking water [15, 16]. These may enter the digestive system through the upper respiratory tract, or as dust settles on the surface of the body, and eventually colonise neonatal intestines. In this paper, *S. epidermidis* was selected for traceability of ARGs by MSLT because of its high abundance, isolation and resistance rate in the above samples. MLST analyses only a small percentage of the genome, which means that it can not distinguish strains accurately enough. However, we tried to make up for this deficiency by comparing the drug resistance gene and drug resistance phenotype of isolated *Staphylococcus epidermidis* of same ST number. And only the *Staphylococcus epidermidis* with the same of ST number, ARGs and drug resistance phenotype considered similar in terms of the criteria listed.

Data analysis, McNemar’s chi square tests and pearson correlation, require for a certain sample size. However, related studies recruited a small sample size due to ethical and compliance barriers. Representative AR gene pools in the study involved 16 healthy infant vaginally delivered and two mother, including *tetM*, *ermB*, *sul2*, and *blaTEM* were detected in infant subjects [20], consistant with our results. The study, 16 mother-infant pairs involved, used a metagenomic approach to determine the diversity of ARGs between fecal samples from the mothers and the infants vaginally delivered at the ages of 1 and 6 months [27]. Infants shared 20% of their ARGs in their guts with breast milk of their mothers correspondingly, our result shown 100% were shared, though only seven drug resistance genes were involved. There are not enough samples for the studies above that it is inadequate to use the method of data statistics to explore the source, and only use description by shared ARGs instead.

During the experimental design, we fully considered the various environmental factors that newborns may be exposed to after birth, including breast milk, air and drinking water. The detection frequencies, antibiotic resistance spectra, and coincidence rate for ARGs in neonatal faeces were highly consistent with those in colostrum samples. *S. epidermidis* was also mainly derived from colostrum. Interestingly, *ampC* in neonatal faeces was significantly higher than in colostrum, and detection of *ampC* was highest in ward air. The origin of *S. epidermidis* in neonatal faeces was also consistent with these results, suggesting that this gene may be derived from air as well as colostrum.

In conclusion, this study demonstrated that intestinal ARGs in neonates are acquired immediately after birth and remained stable in 30 days, mainly from the mother’s colostrum, by excluding acquisition through the birth canal by careful experimental design. A few ARGs, such as *ampC*, may also be derived from hospital ward air.

## Supplementary Information

**Additional file 1: Table S1.** Primers of ARGs for conventional PCR. **Table S2.** Primers of ARGs and 16S rRNA for quantitative PCR. **Table S3.** Standard curves of ARGs and 16S rRNA for quantitative PCR. **Table S4.** Concentration and purity of standard plasmid of ARGs. **Table S5.** Primers of housekeeping genes of *s. epidermidis* [13]. **Table S6.** Effect of gender on thefrequencies of fecal resistance genes in newborns. **Table S7.** Difference and association of the frequencies of blaTEM in colostrums and the feces on the third day of newborns. **Table S8.** Difference and association of the frequencies of ampC in colostrums and the feces on the third day of newborns. **Table S9.** Difference and association of the frequencies of tetM in colostrums and the feces on the third day of newborns. **Table S10.** Difference and association of the frequencies of aac(6′)-Ib in colostrums and the feces on the third day of newborns. **Table S11.** Difference and association of the frequencies of ermB in colostrums and the feces on the third day of newborns. **Table S12.** Difference and association of the frequencies of sul2 in colostrums and the feces on the third day of newborns. **Table S13.** Difference and association of the frequencies of mecA in colostrums and the feces on the third day of newborns. **Figure S1.** The median of ARGs amount in the feces of six newborns on 3th day, 7th day and 30th day, respectively. **Table S14.** ST number of *Staphylococcus epidermidis* isolated from colostrum. **Table S15.** ST number of *Staphylococcus epidermidis* isolated from faeces on the third day. **Table S16.** ST number of *Staphylococcus epidermidis* isolated from ward air. **Table S17.** Resistance phenotypes and ARGs of *Staphylococcus epidermidis* isolated from colostrum. **Table S18.** Resistance phenotypes and ARGs of *Staphylococcus epidermidis* isolated from faeces on third day. **Table S19.** Resistance phenotypes and ARGs of *Staphylococcus epidermidis* isolated from ward air.

## Data Availability

All data generated or analysed during this study are included in this published article.
