# Supplementary material for: Antibiotic resistance genes in gut of breast-fed neonates born by caesarean section originate from breast milk and hospital ward air
Source: BMC Microbiol. 2022 Jan 29;22:36. doi: 10.1186/s12866-022-02447-8 (PMC8800334; doi:10.1186/s12866-022-02447-8)
Supplement: Supplementary file 1 — Additional file 1: Table S1. Primers of ARGs for conventional PCR. Table S2. Primers of ARGs and 16S rRNA for quantitative PCR. Table S3. Standard curves of ARGs and 16S rRNA for quantitative PCR. Table S4. Concentration and purity of standard plasmid of ARGs. Table S5. Primers of housekeeping genes of s. epidermidis [13]. Table S6. Effect of gender on thefrequencies of fecal resistance genes in newborns. Table S7. Difference and association of the frequencies of blaTEM in colostrums and the feces on the third day of newborns. Table S8. Difference and association of the frequencies of ampC in colostrums and the feces on the third day of newborns. Table S9. Difference and association of the frequencies of tetM in colostrums and the feces on the third day of newborns. Table S10. Difference and association of the frequencies of aac(6′)-Ib in colostrums and the feces on the third day of newborns. Table S11. Difference and association of the frequencies of ermB in colostrums and the feces on the third day of newborns. Table S12. Difference and association of the frequencies of sul2 in colostrums and the feces on the third day of newborns. Table S13. Difference and association of the frequencies of mecA in colostrums and the feces on the third day of newborns. Figure S1. The median of ARGs amount in the feces of six newborns on 3th day, 7th day and 30th day, respectively. Table S14. ST number of Staphylococcus epidermidis isolated from colostrum. Table S15. ST number of Staphylococcus epidermidis isolated from faeces on the third day. Table S16. ST number of Staphylococcus epidermidis isolated from ward air. Table S17. Resistance phenotypes and ARGs of Staphylococcus epidermidis isolated from colostrum. Table S18. Resistance phenotypes and ARGs of Staphylococcus epidermidis isolated from faeces on third day. Table S19. Resistance phenotypes and ARGs of Staphylococcus epidermidis isolated from ward air. [file 12866_2022_2447_MOESM1_ESM.docx]

**SUPPLEMENTARY APPENDIX**

**1. Results of detection for ARGs**

**1.1 *Primers of ARGs for conventional PCR***

Table S1 Primers of ARGs for conventional PCR

| Genes | Sequence（5’-3’） | Size of amplicon(bp) | Reference |
| --- | --- | --- | --- |
| *tetM* | F:GTTAAATAGTGTTCTTGGAG  R: CTAAGATATGGCTCTAACAA | 657 | [[1](#_ENREF_1)] |
| *mecA* | F: TGGCATTCGTGTCACAATCG  R: CTGGAACTTGTTGAGGAGAG | 304 | [[2](#_ENREF_2)] |
| *blaTEM* | F: ATTTCCGTGTCGCCCTTATTC  R:CGTTCATCCATAGTTGCCTGACCGTTCATCCATAGTTGCCTGAC | 800 | [[3](#_ENREF_3)] |
| *ampC* | F: CCTCTTGCTCCACATTTGCT  R: ACAACGTTTGCTGTGTGACG | 189 | [[4](#_ENREF_4)] |
| *ermB* | F: TGGTATTCCAAATGCGTAATG  R: CTGTGGTATGGCGGGTAAGT | 745 | [[5](#_ENREF_5)] |
| *sul2* | F: GCAGGCGCGTAAGCTGA  R: GGCTCGTGTGTGCGGATG | 657 | [[6](#_ENREF_6)] |
| *aac(6’)-Ib* | F: ATGACCTTGCGATGCTCTATGA  R: CGAATGCCTGGCGTGTTT | 486 | [[7](#_ENREF_7)] |
| *BlaNDM-1* | F:CTTCCAACGGTTTGATCGTC  R:ATTGGCATAAGTCGCAATCC | 206 | [[8](#_ENREF_8)] |
| *mcr-1* | F:AGTCCGTTTGTTCTTGTGGC  R:AGATCCTTGGTCTCGGCTTG | 320 | [[9](#_ENREF_9)] |

**1.2 *Conditions for conventional PCR***

DNA amplification was carried out using the following conditions,*tetM*: a 5 min initial denaturing step at 94°C followed by 35 cycles of 94°C for 1 min, 55°C for 1 min and 72°C for 1.5 min and a final extension step at 72°C for 5min; *mecA*: a 5 min initial denaturing step at 94°C followed by 35 cycles of 94°C for 30s, 56°C for 1 min and 72°C for 50s and a final extension step at 72°C for 5min; *blaTEM*: a 5 min initial denaturing step at 94°C followed by 35 cycles of 94°C for 45s, 56°C for 45s and 72°C for 1.5min and a final extension step at 72°C for 5min; *ampC*: a 5 min initial denaturing step at 94°C followed by 35 cycles of 94°C for 45s, 53°C for 30s and 72°C for 40s and a final extension step at 72°C for 5min; *ermB*: a 5 min initial denaturing step at 94°C followed by 35 cycles of 94°C for 40s, 50°C for 40s and 72°C for 1.5min and a final extension step at 72°C for 5min; *sul2*: a 5 min initial denaturing step at 94°C followed by 35 cycles of 94°C for 50s, 57°C for 45s and 72°C for 30s and a final extension step at 72°C for 5min; *aac(6’)-Ib*: a 5 min initial denaturing step at 94°C followed by 35 cycles of 94°C for 50s, 57°C for 40s and 72°C for 50s and a final extension step at 72°C for 5min;*BlaNDM-1*: a 5 min initial denaturing step at 94°C followed by 35 cycles of 94°C for 45s, 53°C for 30s and 72°C for 40s and a final extension step at 72°C for 5min;*mcr-1*: a 5 min initial denaturing step at 94°C followed by 35 cycles of 94°C for 30s, 56°C for 1 min and 72°C for 50s and a final extension step at 72°C for 5min.

**1.3 *Primers and standard curveof ARGs and 16S rRNA for quantitative PCR***

Table S2 Primers of ARGs and 16S rRNA for quantitative PCR

| Genes | Sequence（5’-3’） | Size of amplicon(bp) | Reference |
| --- | --- | --- | --- |
| *tetM* | F:TAATATTGGAGTTTTAGCTCATGTTGATG  R:CCTCTCTGACGTTCTAAAAGCGTATTAT | 147 | [[10](#_ENREF_10)] |
| *mecA* | F: TGGCATTCGTGTCACAATCG  R: CTGGAACTTGTTGAGGAGAG | 304 | [[2](#_ENREF_2)] |
| *blaTEM* | F: AGCATCTTACGGATGGCATGA  R: TCCTCCGATCGTTGTCAGAAGT | 103 | [[10](#_ENREF_10)] |
| *ampC* | F: CCTCTTGCTCCACATTTGCT  R: ACAACGTTTGCTGTGTGACG | 189 | [[4](#_ENREF_4)] |
| *ermB* | F: TAAAGGGCATTTAACGACGAAACT  R:TTTATACCTCTGTTTGTTAGGGAATTGAA | 172 | [[10](#_ENREF_10)] |
| *sul2* | F:TCCGGTGGAGGCCGGTATCTGG  R: CGGGAATGCCATCTGCCTTGAG | 105 | [[10](#_ENREF_10)] |
| *aac(6’)-Ib* | F: AGAAGCCGCCCGACACTT  R: GCTCTCCATTCAGCATTGCA | 101 | [[11](#_ENREF_11)] |
| *BlaNDM-1* | F:CTTCCAACGGTTTGATCGTC  R:ATTGGCATAAGTCGCAATCC | 206 | [[8](#_ENREF_8)] |
| *mcr-1* | F:AGTCCGTTTGTTCTTGTGGC  R:AGATCCTTGGTCTCGGCTTG | 320 | [[9](#_ENREF_9)] |
| *16S rRNA* | F: CGGTGAATACGTTCYCGG  R: GGWTACCTTGTTACGACTT | 142 | [[12](#_ENREF_12)] |

Table S3 Standard curves of ARGs and 16S rRNA for quantitative PCR

| Genes | standard curve | r^2^ | Efficiency | Detectionlimit（copies/μL） | NTC Cq |
| --- | --- | --- | --- | --- | --- |
| *tetM* | y=-3.37x+40.97 | 0.998 | 0.980 | 59 | none |
| *mecA* | y=-3.60x+43.35 | 0.998 | 0.896 | 49 | none |
| *blaTEM* | y=-3.05x+41.39 | 0.998 | 0.980 | 38 | 38.34 |
| *ampC* | y=-3.52x+36.21 | 0.999 | 0.923 | 46 | 36.23 |
| *ermB* | y=-3.63x+36.14 | 0.999 | 0.886 | 40 | none |
| *sul2* | y=-3.63x+37.71 | 0.998 | 0.886 | 51 | 36.89 |
| *aac(6’)-Ib* | y=-3.31x+41.28 | 0.998 | 1.005 | 37 | 39.38 |
| *16S* | y=-3.29x+36.37 | 0.999 | 1.013 | 42 | 37.44 |

The template DNA is the DNA extracted from the positive fecal sample, and the template of 16SrDNA is the 16SrDNA fragment of E. coli 25922. The mixed reaction solution is prepared according to the following reagent groups, with a total of 50μL system for PCR reaction. The conventional PCR reaction conditions of each drug resistance gene are as follows: a 5 min initial denaturing step at 94°C followed by 35 cycles of 94°C for 30 seconds, 56°C for 30 seconds and 72°C for 1 min and a final extension step at 72°C for 5min. Then The PCR product gel of drug-resistant gene was recovered and purified with gel Purification Kit (TIANGEN, DP209). The purity and concentration of the target product after recovery and purification were determined by protein nucleic acid analyzer (GeneQuant1300, GE).

Construction of recombinant plasmid of drug resistance gene with the pMD19-T Vector. Extraction of standard quality granules and determination of purity and concentration. Plasmids were extracted by plasmid DNA Extraction Kit (Tiangen). The extracted plasmids were diluted 10 times with enzyme-free water, and the purity and concentration of the extracted plasmid standard were determined by nucleic acid protein analyzer 1300. The size of pMD19-T plasmid selected in this experiment was 2692bp. The purity a260 / A280 and concentration of standard quality granules extracted from drug-resistant genes in this experiment are shown in Table 2-3:

| ARGs | *blaTEM* | *ampC* | *mecA* | *aac(6’)-Ib* | *ermB* | *sul2* | *tetM* |
| --- | --- | --- | --- | --- | --- | --- | --- |
| Concentration(10^10^copies/μL) | 3.8 | 4.6 | 4.9 | 3.7 | 4.0 | 5.1 | 5.9 |
| A260/A280 | 1.833 | 1.857 | 1.796 | 1.829 | 1.883 | 1.841 | 1.871 |

Table S4 Concentration and purity of standard plasmid of ARGs

The concentration of 16SrDNA extraction was 4.2×10^10^copies/ μL. The purity is 1.738. The concentration and purity meet the requirements of the standard, and the next standard curve PCR reaction can be carried out.The reaction system of quantitative PCR of drug resistance gene was 20 μL. Using ABI7300 quantitative PCR instrument, the template is ARGs and 16SrDNA standard vector. After gradient dilution of the standard, each gradient plasmid was used as the template for PCR reaction and draw the standard curve. Quantitative PCR reaction conditions: 50 ℃ for 2 min, 95 ℃ for 10 min, 40 cycles (95 ℃ for 15 s, 60 ℃ for 1 min).

The detection limits were generated by diluting the concentration gradient of pMD19-T plasmid, ARGs involved, to 10^0^ copies/μL, 3 parallel samples were set to ensure the Cqs were limited to one standard deviation (Table S4).

**1.4 *Identity of staphylococcus epidermidis by Multi-locus Sequence Typing (MLST)***

Table S5 Primers of housekeeping genes of *s. epidermidis[*[*13*](#_ENREF_13)*]*

| Genes | Sequence（5’-3’） | Size of amplicon(bp) |
| --- | --- | --- |
| *arcC* | F:TGTGATGAGCACGCTACCGTTAG  R:TCCAAGTAAACCCATCGGTCTG | 508 |
| *aroE* | F: CATTGGATTACCTCTTTGTTCAGC  R: CAAGCGAAATCTGTTGGGG | 459 |
| *gtr* | F:CAGCCAATTCTTTTATGACTTTT  R:GTGATTAAAGGTATTGATTTGAAT | 508 |
| *mutS* | F:GATATAAGAATAAGGGTTGTGAA  R:GTAATCGTCTCAGTTATCATGTT | 608 |
| *pyrR* | F: GTTACTAATACTTTTGCTGTGTTT  R:GTAGAATGTAAAGAGACTAAAATGAA | 851 |
| *TpiA* | F:ATCCAATTAGACGCTTTAGTAAC  R: TTAATGATGCGCCACCTACA | 592 |
| *yqiL* | F:CACGCATAGTATTAGCTGAAG  R:CTAATGCCTTCATCTTGAGAAATAA | 658 |

**1.5 *The conditions for conventional PCR of housekeeping genes of s. epidermidis***

DNA amplification was carried out using the following conditions: a 5 min initial denaturing step at 94°C followed by 35 cycles of 94°C for 40s, 58°C for 40s and 72°C for 1.5 min and a final extension step at 72°C for 5min.

**1.6 *Effect of gender on the frequencies of fecal resistance genes in newborns***

Table S6 Effect of gender on thefrequencies of fecal resistance genes in newborns

| ARGs | χ^2^ | P |
| --- | --- | --- |
| *blaTEM* | 0.058 | 0.809 |
| *ampC* | 3.105 | 0.078 |
| *tetM* | 0.362 | 0.547 |
| *aac6ib* | 0.035 | 0.852 |
| *Ermb* | 0.164 | 0.686 |
| *sul2* | 0.032 | 0.857 |
| *mecA* | 3.586 | 0.058 |

***1.7 Difference of the frequencies of ARGs in colostrums and the feces on the third day of newborns***

|  | | | | |
| --- | --- | --- | --- | --- |
| Table S7 Difference and association of the frequencies of blaTEM in colostrums and the feces on the third day of newborns | | | | |
|  | | feces | | Total |
|  |  | + | ﹣ |  |
| colostrum | + | 11 | 9 | 20 |
|  | ﹣ | 10 | 52 | 62 |
| Total | | 21 | 61 | 82 |

McNemar’s chi square test, p >0.05; Pearson correlation, p <0.05

Table S8 Difference and association of the frequencies of ampC in colostrums and the feces on the third day of newborns

|  | | feces | | Total |
| --- | --- | --- | --- | --- |
|  |  | + | ﹣ |  |
| colostrum | + | 9 | 4 | 13 |
|  | ﹣ | 14 | 55 | 69 |
| Total | | 23 | 59 | 82 |

McNemar’s chi square test, p <0.05; Pearson correlation, p <0.05

|  | | | | |
| --- | --- | --- | --- | --- |
| Table S9 Difference and association of the frequencies of tetM in colostrums and the feces on the third day of newborns | | | | |
|  | | feces | | Total |
|  |  | + | ﹣ |  |
| colostrum | + | 11 | 8 | 19 |
|  | ﹣ | 7 | 56 | 63 |
| Total | | 18 | 64 | 82 |

McNemar’s chi square test, p >0.05; Pearson correlation, p <0.05

|  | | | | |
| --- | --- | --- | --- | --- |
| Table S10 Difference and association of the frequencies of aac(6’)-Ib in colostrums and the feces on the third day of newborns | | | | |
|  | | feces | | Total |
|  |  | + | ﹣ |  |
| colostrum | + | 7 | 7 | 14 |
|  | ﹣ | 7 | 61 | 68 |
| Total | | 14 | 68 | 82 |

McNemar’s chi square test, p >0.05; Pearson correlation, p <0.05

|  | | | | |
| --- | --- | --- | --- | --- |
| Table S11 Difference and association of the frequencies of ermB in colostrums and the feces on the third day of newborns | | | | |
|  | | feces | | Total |
|  |  | + | ﹣ |  |
| colostrum | + | 13 | 8 | 21 |
|  | ﹣ | 11 | 50 | 61 |
| Total | | 24 | 58 | 82 |

McNemar’s chi square test, p >0.05; Pearson correlation, p <0.05

|  | | | | |
| --- | --- | --- | --- | --- |
| Table S12 Difference and association of the frequencies of sul2 in colostrums and the feces on the third day of newborns | | | | |
|  | | feces | | Total |
|  |  | + | ﹣ |  |
| colostrum | + | 7 | 10 | 17 |
|  | ﹣ | 7 | 58 | 65 |
| Total | | 14 | 68 | 82 |

McNemar’s chi square test, p >0.05; Pearson correlation, p <0.05

|  | | | | |
| --- | --- | --- | --- | --- |
| Table S13 Difference and association of the frequencies of mecA in colostrums and the feces on the third day of newborns | | | | |
|  | | feces | | Total |
|  |  | + | ﹣ |  |
| colostrum | + | 19 | 11 | 30 |
|  | ﹣ | 18 | 34 | 52 |
| Total | | 37 | 45 | 82 |

McNemar’s chi square test, p >0.05; Pearson correlation, p <0.05

***1.8 The median of ARGs amount in the feces of six newborns on 3th day, 7th day and 30th day, respectively.***


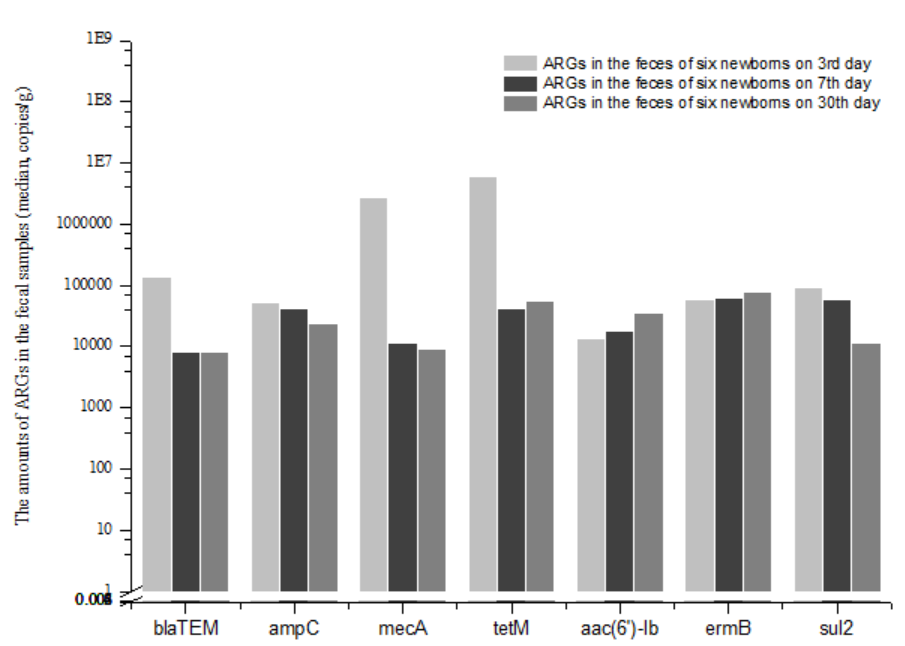


Figure S1. The median of ARGs amount in the feces of six newborns on 3th day, 7th day and 30th day, respectively.

**2. Results of detection for S. epidermidis**

***2.1 STnumber of Staphylococcus epidermidis isolated from colostrum、faeces on third day、ward air***

Table S14 ST number of Staphylococcus epidermidis isolated from colostrum

| subjects | ST number | arcC | aroE | gtr | mutS | pyr | tpi | yqiL |
| --- | --- | --- | --- | --- | --- | --- | --- | --- |
| mother1 | ST2 | 7 | 1 | 2 | 2 | 4 | 1 | 1 |
| mother2* | ST2 | 7 | 1 | 2 | 2 | 4 | 1 | 1 |
| mother3 | ST5 | 1 | 1 | 1 | 2 | 2 | 1 | 1 |
| mother4 | ST2 | 7 | 1 | 2 | 2 | 4 | 1 | 1 |
| mother5 | ST23 | 7 | 1 | 2 | 1 | 3 | 3 | 1 |
| mother6* | ST6 | 1 | 1 | 2 | 2 | 2 | 1 | 1 |
| mother7* | ST2 | 7 | 1 | 2 | 2 | 4 | 1 | 1 |
| mother8* | ST6 | 1 | 1 | 2 | 2 | 2 | 1 | 1 |
| mother9 | ST2 | 7 | 1 | 2 | 2 | 4 | 1 | 1 |
| mother10 | ST10 | 1 | 1 | 1 | 1 | 3 | 1 | 1 |
| mother11* | ST23 | 7 | 1 | 2 | 1 | 3 | 3 | 1 |
| mother12 | ST2 | 7 | 1 | 2 | 2 | 4 | 1 | 1 |
| mother13* | ST258 | 19 | 15 | 5 | 5 | 3 | 9 | 10 |
| mother14 | ST422 | 1 | 1 | 1 | 2 | 2 | 1 | 16 |
| mother15* | ST34 | 1 | 1 | 2 | 2 | 1 | 13 | 3 |
| mother16* | ST23 | 7 | 1 | 2 | 1 | 3 | 3 | 1 |
| mother17* | ST2 | 7 | 1 | 2 | 2 | 4 | 1 | 1 |
| mother18 | ST342 | 7 | 1 | 2 | 5 | 4 | 1 | 1 |
| mother19 | ST156 | 1 | 2 | 6 | 2 | 1 | 1 | 11 |
| mother20 | ST231 | 7 | 1 | 2 | 26 | 3 | 3 | 1 |
| mother21 | ST5 | 1 | 1 | 1 | 2 | 2 | 1 | 1 |
| mother22* | ST2 | 7 | 1 | 2 | 2 | 4 | 1 | 1 |
| mother23* | ST456 | 1 | 2 | 2 | 1 | 1 | 1 | 8 |
| mother24 | ST10 | 1 | 1 | 1 | 1 | 3 | 1 | 1 |
| mother25* | ST144 | 7 | 1 | 12 | 2 | 4 | 1 | 1 |
| mother26 | ST24 | 3 | 1 | 5 | 5 | 11 | 16 | 11 |
| mother27* | ST2 | 7 | 1 | 2 | 2 | 4 | 1 | 1 |
| mother28* | ST5 | 1 | 1 | 1 | 2 | 2 | 1 | 1 |
| mother29 | ST77 | 23 | 1 | 1 | 2 | 2 | 1 | 1 |
| mother30* | ST166 | 1 | 1 | 2 | 2 | 2 | 1 | 3 |
| mother31* | ST10 | 1 | 1 | 1 | 1 | 3 | 1 | 1 |
| mother32 | ST334 | 1 | 1 | 33 | 6 | 2 | 16 | 1 |
| mother33 | ST54 | 1 | 1 | 2 | 2 | 4 | 1 | 1 |
| mother34* | ST2 | 7 | 1 | 2 | 2 | 4 | 1 | 1 |
| mother35 | ST142 | 2 | 1 | 2 | 2 | 2 | 1 | 1 |
| mother36 | ST10 | 1 | 1 | 1 | 1 | 3 | 1 | 1 |
| mother37 | ST536 | 1 | 5 | 1 | 2 | 2 | 1 | 1 |
| mother38* | ST259 | 12 | 1 | 2 | 1 | 2 | 1 | 1 |
| mother39 | ST2 | 7 | 1 | 2 | 2 | 4 | 1 | 1 |
| mother40* | ST492 | 28 | 49 | 15 | 7 | 43 | 34 | 4 |
| mother41* | ST23 | 7 | 1 | 2 | 1 | 3 | 3 | 1 |
| mother42 | ST5 | 1 | 1 | 1 | 2 | 2 | 1 | 1 |
| mother43 | ST2 | 7 | 1 | 2 | 2 | 4 | 1 | 1 |
| mother44 | ST144 | 7 | 1 | 12 | 2 | 4 | 1 | 1 |
| mother45* | ST342 | 7 | 1 | 2 | 5 | 4 | 1 | 1 |
| mother46 | ST276 | 34 | 10 | 1 | 5 | 10 | 16 | 21 |
| mother47 | ST2 | 7 | 1 | 2 | 2 | 4 | 1 | 1 |
| mother48 | ST5 | 1 | 1 | 1 | 2 | 2 | 1 | 1 |
| mother49* | ST24 | 3 | 1 | 5 | 5 | 11 | 16 | 11 |
| mother50* | ST2 | 7 | 1 | 2 | 2 | 4 | 1 | 1 |
| mother51 | ST37 | 18 | 1 | 5 | 5 | 11 | 4 | 20 |
| mother52 | ST462 | 12 | 10 | 5 | 5 | 7 | 16 | 21 |
| mother53 | ST10 | 1 | 1 | 1 | 1 | 3 | 1 | 1 |
| mother54* | ST322 | 3 | 34 | 9 | 5 | 33 | 28 | 12 |
| mother55 | ST65 | 1 | 19 | 17 | 4 | 9 | 10 | 2 |
| mother56 | ST2 | 7 | 1 | 2 | 2 | 4 | 1 | 1 |
| mother57 | ST2 | 7 | 1 | 2 | 2 | 4 | 1 | 1 |
| mother58* | ST5 | 1 | 1 | 1 | 2 | 2 | 1 | 1 |
| mother59 | ST2 | 7 | 1 | 2 | 2 | 4 | 1 | 1 |
| mother60 | ST10 | 1 | 1 | 1 | 1 | 3 | 1 | 1 |
| mother61 | ST322 | 3 | 34 | 9 | 5 | 33 | 28 | 12 |
| mother62 | ST2 | 7 | 1 | 2 | 2 | 4 | 1 | 1 |
| mother63* | ST54 | 1 | 1 | 2 | 2 | 4 | 1 | 1 |
| mother64 | ST10 | 1 | 1 | 1 | 1 | 3 | 1 | 1 |
| mother65 | ST231 | 7 | 1 | 2 | 26 | 3 | 3 | 1 |
| mother66 | ST10 | 1 | 1 | 1 | 1 | 3 | 1 | 1 |
| mother67 | ST2 | 7 | 1 | 2 | 2 | 4 | 1 | 1 |
| mother68* | ST422 | 1 | 1 | 1 | 2 | 2 | 1 | 16 |
| mother69 | ST334 | 1 | 1 | 33 | 6 | 2 | 16 | 1 |
| mother70 | ST5 | 1 | 1 | 1 | 2 | 2 | 1 | 1 |
| mother71* | ST77 | 23 | 1 | 1 | 2 | 2 | 1 | 1 |
| mother72 | ST2 | 7 | 1 | 2 | 2 | 4 | 1 | 1 |
| mother73 | ST10 | 1 | 1 | 1 | 1 | 3 | 1 | 1 |
| mother74 | ST2 | 7 | 1 | 2 | 2 | 4 | 1 | 1 |
| mother75 | ST65 | 1 | 19 | 17 | 4 | 9 | 10 | 2 |
| mother76 | ST2 | 7 | 1 | 2 | 2 | 4 | 1 | 1 |
| mother77* | ST456 | 1 | 2 | 2 | 1 | 1 | 1 | 8 |
| mother78 | ST5 | 1 | 1 | 1 | 2 | 2 | 1 | 1 |
| mother79 | ST65 | 1 | 19 | 17 | 4 | 9 | 10 | 2 |
| mother80 | ST2 | 7 | 1 | 2 | 2 | 4 | 1 | 1 |
| mother81 | ST2 | 7 | 1 | 2 | 2 | 4 | 1 | 1 |
| mother82* | ST10 | 1 | 1 | 1 | 1 | 3 | 1 | 1 |

*“*” Staphylococcus epidermidis isolated from colostrum is different with that from faeces on the third day about ST number correspondingly.*

Table S15 ST number of Staphylococcus epidermidis isolated from faeces on the third day

| subjects | ST number | arcC | aroE | gtr | mutS | pyr | tpi | yqiL |
| --- | --- | --- | --- | --- | --- | --- | --- | --- |
| newborn1 | ST2 | 7 | 1 | 2 | 2 | 4 | 1 | 1 |
| newborn2* | ST5 | 1 | 1 | 1 | 2 | 2 | 1 | 1 |
| newborn3 | ST5 | 1 | 1 | 1 | 2 | 2 | 1 | 1 |
| newborn4 | ST2 | 7 | 1 | 2 | 2 | 4 | 1 | 1 |
| newborn5 | ST23 | 7 | 1 | 2 | 1 | 3 | 3 | 1 |
| newborn6* | ST2 | 7 | 1 | 2 | 2 | 4 | 1 | 1 |
| newborn7* | ST66 | 12 | 3 | 5 | 5 | 7 | 14 | 11 |
| newborn8* | ST5 | 1 | 1 | 1 | 2 | 2 | 1 | 1 |
| newborn9 | ST2 | 7 | 1 | 2 | 2 | 4 | 1 | 1 |
| newborn10 | ST2 | 7 | 1 | 2 | 2 | 4 | 1 | 1 |
| newborn11* | ST5 | 1 | 1 | 1 | 2 | 2 | 1 | 1 |
| newborn12 | ST2 | 7 | 1 | 2 | 2 | 4 | 1 | 1 |
| newborn13* | ST2 | 7 | 1 | 2 | 2 | 4 | 1 | 1 |
| newborn14 | ST422 | 1 | 1 | 1 | 2 | 2 | 1 | 16 |
| newborn15* | ST66 | 12 | 3 | 5 | 5 | 7 | 14 | 11 |
| newborn16* | ST2 | 7 | 1 | 2 | 2 | 4 | 1 | 1 |
| newborn17* | ST5 | 1 | 1 | 1 | 2 | 2 | 1 | 1 |
| newborn18 | ST342 | 7 | 1 | 2 | 5 | 4 | 1 | 1 |
| newborn19 | ST156 | 1 | 2 | 6 | 2 | 1 | 1 | 11 |
| newborn20 | ST231 | 7 | 1 | 2 | 26 | 3 | 3 | 1 |
| newborn21 | ST5 | 1 | 1 | 1 | 2 | 2 | 1 | 1 |
| newborn22* | ST10 | 1 | 1 | 1 | 1 | 3 | 1 | 1 |
| newborn23* | ST2 | 7 | 1 | 2 | 2 | 4 | 1 | 1 |
| newborn24 | ST10 | 1 | 1 | 1 | 1 | 3 | 1 | 1 |
| newborn25* | ST2 | 7 | 1 | 2 | 2 | 4 | 1 | 1 |
| newborn26 | ST24 | 3 | 1 | 5 | 5 | 11 | 16 | 11 |
| newborn27* | ST2 | 7 | 1 | 2 | 2 | 4 | 1 | 1 |
| newborn28* | ST142 | 2 | 1 | 2 | 2 | 2 | 1 | 1 |
| newborn29 | ST77 | 23 | 1 | 1 | 2 | 2 | 1 | 1 |
| newborn30* | ST24 | 3 | 1 | 5 | 5 | 11 | 16 | 11 |
| newborn31* | ST2 | 7 | 1 | 2 | 2 | 4 | 1 | 1 |
| newborn32 | ST334 | 1 | 1 | 33 | 6 | 2 | 16 | 1 |
| newborn33 | ST54 | 1 | 1 | 2 | 2 | 4 | 1 | 1 |
| newborn34* | ST5 | 1 | 1 | 1 | 2 | 2 | 1 | 1 |
| newborn35 | ST142 | 2 | 1 | 2 | 2 | 2 | 1 | 1 |
| newborn36 | ST10 | 1 | 1 | 1 | 1 | 3 | 1 | 1 |
| newborn37 | ST536 | 1 | 5 | 1 | 2 | 2 | 1 | 1 |
| newborn38* | ST10 | 1 | 1 | 1 | 1 | 3 | 1 | 1 |
| newborn39 | ST2 | 7 | 1 | 2 | 2 | 4 | 1 | 1 |
| newborn40* | ST2 | 7 | 1 | 2 | 2 | 4 | 1 | 1 |
| newborn41* | ST462 | 12 | 10 | 5 | 5 | 7 | 16 | 21 |
| newborn42 | ST5 | 1 | 1 | 1 | 2 | 2 | 1 | 1 |
| newborn43 | ST2 | 7 | 1 | 2 | 2 | 4 | 1 | 1 |
| newborn44 | ST144 | 7 | 1 | 12 | 2 | 4 | 1 | 1 |
| newborn45* | ST2 | 7 | 1 | 2 | 2 | 4 | 1 | 1 |
| newborn46 | ST276 | 34 | 10 | 1 | 5 | 10 | 16 | 21 |
| newborn47 | ST2 | 7 | 1 | 2 | 2 | 4 | 1 | 1 |
| newborn48 | ST5 | 1 | 1 | 1 | 2 | 2 | 1 | 1 |
| newborn49* | ST231 | 7 | 1 | 2 | 26 | 3 | 3 | 1 |
| newborn50* | ST10 | 1 | 1 | 1 | 1 | 3 | 1 | 1 |
| newborn51 | ST37 | 18 | 1 | 5 | 5 | 11 | 4 | 20 |
| newborn52 | ST462 | 12 | 10 | 5 | 5 | 7 | 16 | 21 |
| newborn53 | ST10 | 1 | 1 | 1 | 1 | 3 | 1 | 1 |
| newborn54* | ST5 | 1 | 1 | 1 | 2 | 2 | 1 | 1 |
| newborn55 | ST65 | 1 | 19 | 17 | 4 | 9 | 10 | 2 |
| newborn56 | ST2 | 7 | 1 | 2 | 2 | 4 | 1 | 1 |
| newborn57 | ST2 | 7 | 1 | 2 | 2 | 4 | 1 | 1 |
| newborn58* | ST10 | 1 | 1 | 1 | 1 | 3 | 1 | 1 |
| newborn59 | ST2 | 7 | 1 | 2 | 2 | 4 | 1 | 1 |
| newborn60 | ST10 | 1 | 1 | 1 | 1 | 3 | 1 | 1 |
| newborn61 | ST322 | 3 | 34 | 9 | 5 | 33 | 28 | 12 |
| newborn62 | ST2 | 7 | 1 | 2 | 2 | 4 | 1 | 1 |
| newborn63* | ST5 | 1 | 1 | 1 | 2 | 2 | 1 | 1 |
| newborn64 | ST10 | 1 | 1 | 1 | 1 | 3 | 1 | 1 |
| newborn65 | ST231 | 7 | 1 | 2 | 26 | 3 | 3 | 1 |
| newborn66 | ST10 | 1 | 1 | 1 | 1 | 3 | 1 | 1 |
| newborn67 | ST2 | 7 | 1 | 2 | 2 | 4 | 1 | 1 |
| newborn68* | ST5 | 1 | 1 | 1 | 2 | 2 | 1 | 1 |
| newborn69 | ST334 | 1 | 1 | 33 | 6 | 2 | 16 | 1 |
| newborn70 | ST5 | 1 | 1 | 1 | 2 | 2 | 1 | 1 |
| newborn71* | ST2 | 7 | 1 | 2 | 2 | 4 | 1 | 1 |
| newborn72 | ST2 | 7 | 1 | 2 | 2 | 4 | 1 | 1 |
| newborn73 | ST10 | 1 | 1 | 1 | 1 | 3 | 1 | 1 |
| newborn74 | ST2 | 7 | 1 | 2 | 2 | 4 | 1 | 1 |
| newborn75 | ST65 | 1 | 19 | 17 | 4 | 9 | 10 | 2 |
| newborn76 | ST2 | 7 | 1 | 2 | 2 | 4 | 1 | 1 |
| newborn77* | ST2 | 7 | 1 | 2 | 2 | 4 | 1 | 1 |
| newborn78 | ST5 | 1 | 1 | 1 | 2 | 2 | 1 | 1 |
| newborn79 | ST65 | 1 | 19 | 17 | 4 | 9 | 10 | 2 |
| newborn80 | ST2 | 7 | 1 | 2 | 2 | 4 | 1 | 1 |
| newborn81 | ST2 | 7 | 1 | 2 | 2 | 4 | 1 | 1 |
| newborn82* | ST2 | 7 | 1 | 2 | 2 | 4 | 1 | 1 |

*“*” Staphylococcus epidermidis isolated from faeces on the third day is different with that from colostrum about ST number correspondingly.*

Table S16 ST number of Staphylococcus epidermidis isolated from ward air

| ST number | arcC | aroE | gtr | mutS | pyr | tpi | yqiL |
| --- | --- | --- | --- | --- | --- | --- | --- |
| ST2* | 7 | 1 | 2 | 2 | 4 | 1 | 1 |
| ST2 | 7 | 1 | 2 | 2 | 4 | 1 | 1 |
| ST2* | 7 | 1 | 2 | 2 | 4 | 1 | 1 |
| ST5* | 1 | 1 | 1 | 2 | 2 | 1 | 1 |
| ST10* | 1 | 1 | 1 | 1 | 3 | 1 | 1 |
| ST10* | 1 | 1 | 1 | 1 | 3 | 1 | 1 |
| ST2 | 7 | 1 | 2 | 2 | 4 | 1 | 1 |
| ST6* | 1 | 1 | 2 | 2 | 2 | 1 | 1 |
| ST59* | 2 | 1 | 1 | 1 | 2 | 1 | 1 |
| ST71* | 3 | 1 | 5 | 5 | 3 | 1 | 11 |
| ST2 | 7 | 1 | 2 | 2 | 4 | 1 | 1 |

*“*” Staphylococcus epidermidis isolated from ward air is different with that from faeces on the third day about ST number*

***2.2 Resistance phenotypes and ARGs of Staphylococcus epidermidis isolated from colostrum、faeces on third day、ward air.***

Table S17Resistance phenotypes and ARGs of Staphylococcus epidermidis isolated from colostrum

| subjects | Ampicillin | Tetracycline | Erythromycin | Cefotaxime | Kanamycin | Vancomycin | Ofloxacin | Chloramphenicol | Sulfamethoxazole | Penicillin | blaTEM | ampc | tetm | aac6ib | ermb | sul2 | mecA |
| --- | --- | --- | --- | --- | --- | --- | --- | --- | --- | --- | --- | --- | --- | --- | --- | --- | --- |
| mother1 | ＋ |  |  |  |  |  |  |  |  |  |  |  |  |  |  |  | ＋ |
| mother2* | ＋ |  | ＋ |  |  |  |  |  |  |  | ＋ |  |  |  | ＋ |  | ＋ |
| mother3 |  | ＋ |  |  |  |  | ＋ |  |  |  |  | ＋ | ＋ |  |  | ＋ |  |
| mother4 |  |  | ＋ |  |  |  |  |  |  | ＋ |  |  |  |  |  |  |  |
| mother5 |  |  |  |  |  |  |  |  |  |  |  |  |  |  |  |  |  |
| mother6* |  |  | ＋ | ＋ |  |  |  |  | ＋ |  |  |  |  |  |  |  |  |
| mother7* | ＋ |  |  |  |  |  |  |  |  |  |  |  |  |  |  |  | ＋ |
| mother8* |  |  |  |  |  |  |  |  |  |  | ＋ |  |  |  |  |  |  |
| mother9 | ＋ | ＋ |  |  |  |  |  |  | ＋ |  |  |  |  |  |  |  |  |
| mother10 |  |  |  |  |  |  |  | ＋ |  |  |  |  |  |  |  |  |  |
| mother11* |  |  | ＋ |  |  |  |  |  |  |  |  |  |  |  | ＋ |  |  |
| mother12 |  |  |  | ＋ |  |  | ＋ |  |  |  |  |  |  |  |  |  |  |
| mother13* | ＋ |  |  |  | ＋ |  |  |  | ＋ |  |  |  |  | ＋ |  | ＋ | ＋ |
| mother14 |  | ＋ |  |  |  |  |  |  |  |  |  |  | ＋ |  |  |  |  |
| mother15* |  |  | ＋ |  |  |  |  |  |  | ＋ |  |  |  |  |  |  |  |
| mother16* |  |  |  |  |  |  |  |  |  |  |  |  |  |  |  |  |  |
| mother17* |  |  | ＋ |  |  |  |  |  |  |  |  |  |  |  |  |  |  |
| mother18 |  |  |  |  | ＋ |  | ＋ |  |  |  |  |  |  | ＋ |  |  |  |
| mother19 | ＋ |  |  |  |  |  |  |  |  | ＋ |  |  |  |  |  |  | ＋ |
| mother20 |  |  |  |  |  |  |  |  |  |  |  |  |  |  |  |  |  |
| mother21 |  | ＋ | ＋ |  |  |  |  |  |  |  |  |  |  |  | ＋ |  |  |
| mother22* |  |  |  |  |  |  |  |  |  |  |  |  |  |  |  |  |  |
| mother23* | ＋ |  | ＋ |  |  |  |  |  |  |  |  | ＋ |  |  | ＋ |  | ＋ |
| mother24 |  |  |  |  |  |  |  |  |  |  |  |  |  |  |  |  |  |
| mother25* |  |  |  | ＋ |  |  |  |  | ＋ |  |  |  |  | ＋ |  |  |  |
| mother26 |  |  |  |  | ＋ |  |  |  |  |  |  |  |  |  |  |  |  |
| mother27* |  |  | ＋ |  |  |  |  |  |  |  |  |  |  |  |  |  |  |
| mother28* | ＋ | ＋ |  |  |  |  |  |  |  |  | ＋ |  | ＋ |  |  |  |  |
| mother29 |  |  | ＋ |  |  |  |  | ＋ |  | ＋ |  |  |  |  |  | ＋ |  |
| mother30* |  |  |  |  |  |  |  |  |  | ＋ |  |  |  |  |  |  |  |
| mother31* |  | ＋ |  |  |  |  |  |  |  |  |  |  |  |  |  |  |  |
| mother32 | ＋ |  |  |  |  |  |  |  |  |  |  |  |  |  |  |  |  |
| mother33 |  |  | ＋ |  |  |  |  |  |  |  |  |  |  |  | ＋ |  |  |
| mother34* | ＋ |  |  |  |  |  |  |  |  |  |  |  |  |  |  |  | ＋ |
| mother35 |  |  |  |  |  |  |  | ＋ |  |  |  |  |  |  |  |  |  |
| mother36 |  |  |  |  |  |  | ＋ |  |  |  |  |  |  |  |  |  |  |
| mother37 | ＋ |  |  |  |  |  |  |  | ＋ |  |  |  |  |  |  |  |  |
| mother38* |  |  |  |  |  |  |  |  |  |  |  |  |  |  |  |  |  |
| mother39 | ＋ |  |  |  |  |  |  |  |  |  |  |  |  |  |  |  | ＋ |
| mother40* |  |  |  |  |  |  |  |  | ＋ |  |  |  |  |  |  | ＋ |  |
| mother41* |  |  | ＋ |  | ＋ |  |  |  |  |  |  |  |  |  |  |  |  |
| mother42 | ＋ |  |  |  |  |  |  |  |  |  | ＋ |  |  |  |  |  | ＋ |
| mother43 |  | ＋ | ＋ |  |  |  |  |  |  | ＋ |  |  | ＋ |  | ＋ |  |  |
| mother44 |  |  |  |  |  |  |  |  |  |  |  |  |  |  |  |  |  |
| mother45* | ＋ |  | ＋ |  |  |  |  |  |  |  |  |  |  |  |  |  | ＋ |
| mother46 |  |  |  |  |  |  |  |  |  |  |  |  |  |  |  |  |  |
| mother47 |  | ＋ |  |  |  |  |  |  |  |  |  |  | ＋ |  |  |  | ＋ |
| mother48 | ＋ |  |  |  |  |  |  |  | ＋ |  |  | ＋ |  |  |  |  |  |
| mother49* |  |  |  |  |  |  |  |  |  |  |  |  |  |  |  |  |  |
| mother50* |  |  |  |  |  |  |  |  |  | ＋ |  |  |  |  |  |  |  |
| mother51 | ＋ |  | ＋ |  |  |  |  |  |  |  |  |  |  |  | ＋ |  | ＋ |
| mother52 |  |  | ＋ |  |  |  |  |  |  | ＋ |  |  |  |  |  |  |  |
| mother53 |  |  |  |  | ＋ |  | ＋ |  |  |  |  |  |  | ＋ |  |  |  |
| mother54* |  |  |  |  |  |  |  | ＋ |  |  |  |  |  |  |  |  |  |
| mother55 |  |  |  |  |  |  |  |  |  |  |  |  |  |  |  |  | ＋ |
| mother56 | ＋ |  | ＋ |  |  |  |  |  |  | ＋ | ＋ |  |  |  | ＋ |  | ＋ |
| mother57 |  | ＋ |  |  |  |  |  |  |  |  |  |  | ＋ |  |  |  |  |
| mother58* | ＋ |  |  |  |  |  |  |  |  |  |  |  |  |  |  |  |  |
| mother59 |  |  |  |  |  |  |  |  |  |  |  |  |  |  |  |  |  |
| mother60 |  |  | ＋ |  |  |  |  |  | ＋ |  |  |  |  |  |  | ＋ |  |
| mother61 | ＋ | ＋ |  |  | ＋ |  |  |  |  |  | ＋ | ＋ |  |  |  |  |  |
| mother62 |  |  |  |  |  |  |  |  |  |  |  |  |  |  |  |  |  |
| mother63* | ＋ |  |  | ＋ |  |  |  |  |  | ＋ |  |  |  |  | ＋ |  | ＋ |
| mother64 |  |  |  |  |  |  |  |  |  |  |  |  |  |  |  |  |  |
| mother65 | ＋ |  |  |  |  |  |  |  |  |  |  |  |  |  |  |  |  |
| mother66 |  | ＋ |  |  |  |  |  |  |  |  |  |  | ＋ |  |  |  |  |
| mother67 |  |  |  |  |  |  |  |  |  |  |  | ＋ |  |  |  |  | ＋ |
| mother68* |  |  | ＋ |  |  |  |  |  |  |  |  |  |  |  |  |  |  |
| mother69 |  |  |  | ＋ |  |  |  |  |  |  |  |  |  |  | ＋ | ＋ |  |
| mother70 |  | ＋ |  |  |  |  |  |  | ＋ |  |  |  |  |  |  |  |  |
| mother71* |  |  |  |  |  |  |  |  |  |  |  |  |  |  |  |  |  |
| mother72 |  |  |  |  |  |  |  |  |  | ＋ |  |  |  |  |  |  | ＋ |
| mother73 | ＋ |  |  |  |  |  |  | ＋ |  |  | ＋ |  |  |  |  |  |  |
| mother74 |  |  |  |  |  |  |  |  |  |  |  |  |  |  |  |  |  |
| mother75 | ＋ | ＋ |  |  |  |  |  |  |  |  |  | ＋ |  |  |  |  |  |
| mother76 |  |  |  |  |  |  |  |  |  |  |  |  |  |  | ＋ |  |  |
| mother77* |  |  |  |  |  |  |  |  |  |  |  |  |  |  |  |  |  |
| mother78 |  |  |  |  | ＋ |  |  |  |  |  |  |  | ＋ | ＋ |  |  |  |
| mother79 |  |  |  |  |  |  |  |  | ＋ |  |  |  |  |  |  | ＋ |  |
| mother80 | ＋ |  | ＋ |  |  |  |  |  |  | ＋ |  | ＋ |  |  |  |  | ＋ |
| mother81 |  |  |  |  |  |  |  |  |  |  | ＋ |  |  |  |  |  |  |
| mother82* |  | ＋ |  |  |  |  |  |  |  |  |  |  |  |  |  |  |  |

*“*” Staphylococcus epidermidis isolated from colostrum is different with that from faeces on the third day about resistance phenotypes and ARGs correspondingly.*

*“+” resistance to antibiotics or positive for ARG*

Table S18 Resistance phenotypes and ARGs of Staphylococcus epidermidis isolated from faeces on third day

| subjects | Ampicillin | Tetracycline | Erythromycin | Cefotaxime | Kanamycin | Vancomycin | Ofloxacin | Chloramphenicol | Sulfamethoxazole | Penicillin | blaTEM | ampc | tetm | aac6ib | ermb | sul2 | mecA |
| --- | --- | --- | --- | --- | --- | --- | --- | --- | --- | --- | --- | --- | --- | --- | --- | --- | --- |
| newborn1 | ＋ |  |  |  |  |  |  |  |  |  |  |  |  |  |  |  | ＋ |
| newborn2* | ＋ | ＋ |  |  |  |  |  |  | ＋ |  | ＋ |  | ＋ |  | ＋ |  | ＋ |
| newborn3 |  | ＋ |  |  |  |  | ＋ |  |  |  |  | ＋ | ＋ |  |  | ＋ |  |
| newborn4 |  |  | ＋ |  |  |  |  |  |  | ＋ |  |  |  |  |  |  |  |
| newborn5 |  |  |  |  |  |  |  |  |  |  |  |  |  |  |  |  |  |
| newborn6* | ＋ |  | ＋ |  |  |  |  |  | ＋ |  |  |  |  |  |  | ＋ |  |
| newborn7* | ＋ |  |  |  |  |  |  |  |  |  |  |  |  |  |  |  | ＋ |
| newborn8* |  |  |  |  |  |  |  |  |  |  | ＋ |  |  |  |  |  |  |
| newborn9 | ＋ | ＋ |  |  |  |  |  |  | ＋ |  |  |  |  |  |  |  |  |
| newborn10 |  |  |  |  |  |  |  | ＋ |  |  |  |  |  |  |  |  |  |
| newborn11* | ＋ |  | ＋ | ＋ |  |  |  |  |  |  |  |  |  |  | ＋ |  | ＋ |
| newborn12 |  |  |  | ＋ |  |  | ＋ |  |  |  |  |  |  |  |  |  |  |
| newborn13* | ＋ | ＋ |  |  | ＋ |  |  |  | ＋ |  |  |  | ＋ | ＋ |  |  | ＋ |
| newborn14 |  | ＋ |  |  |  |  |  |  |  |  |  |  | ＋ |  |  |  |  |
| newborn15* | ＋ |  | ＋ |  |  |  | ＋ |  |  | ＋ |  |  |  |  | ＋ |  |  |
| newborn16* |  |  |  | ＋ | ＋ |  |  |  |  |  |  |  |  | ＋ |  |  |  |
| newborn17* |  | ＋ | ＋ |  |  |  |  |  |  |  |  |  |  |  | ＋ |  |  |
| newborn18 |  |  |  |  | ＋ |  | ＋ |  |  |  |  |  |  | ＋ |  |  |  |
| newborn19 | ＋ |  |  |  |  |  |  |  |  | ＋ |  |  |  |  |  |  | ＋ |
| newborn20 |  |  |  |  |  |  |  |  |  |  |  |  |  |  |  |  |  |
| newborn21 |  | ＋ | ＋ |  |  |  |  |  |  |  |  |  |  |  | ＋ |  |  |
| newborn22* |  |  |  |  |  |  |  |  |  | ＋ | ＋ |  |  |  |  |  |  |
| newborn23* | ＋ |  | ＋ |  |  |  |  |  |  |  | ＋ | ＋ |  |  | ＋ |  | ＋ |
| newborn24 |  |  |  |  |  |  |  |  |  |  |  |  |  |  |  |  |  |
| newborn25* |  |  |  | ＋ |  |  |  |  | ＋ |  |  |  |  | ＋ |  |  | ＋ |
| newborn26 |  |  |  |  | ＋ |  |  |  |  |  |  |  |  |  |  |  |  |
| newborn27* |  |  | ＋ |  |  |  |  |  |  |  |  |  |  |  |  |  |  |
| newborn28* | ＋ | ＋ |  |  |  |  |  |  |  |  |  | ＋ | ＋ |  |  |  |  |
| newborn29 |  |  | ＋ |  |  |  |  | ＋ |  | ＋ |  |  |  |  |  | ＋ |  |
| newborn30* |  |  |  |  |  |  | ＋ |  |  | ＋ |  | ＋ |  |  |  |  |  |
| newborn31* |  | ＋ | ＋ |  |  |  |  |  | ＋ |  |  |  |  |  | ＋ |  |  |
| newborn32 | ＋ |  |  |  |  |  |  |  |  |  |  |  |  |  |  |  |  |
| newborn33 |  |  | ＋ |  |  |  |  |  |  |  | ＋ |  |  |  | ＋ |  |  |
| newborn34* | ＋ |  |  |  |  |  |  | ＋ |  |  |  |  |  |  |  |  | ＋ |
| newborn35 |  |  |  |  |  |  |  | ＋ |  |  |  |  |  |  |  |  |  |
| newborn36 |  |  |  |  |  |  | ＋ |  |  |  |  |  |  |  |  |  |  |
| newborn37 | ＋ |  |  |  |  |  |  |  | ＋ |  |  |  |  |  |  |  |  |
| newborn38* |  |  |  | ＋ |  |  |  |  |  |  | ＋ |  |  |  |  |  |  |
| newborn39 | ＋ |  |  |  |  |  |  |  |  |  |  |  |  |  |  |  | ＋ |
| newborn40* |  |  |  | ＋ |  |  |  |  | ＋ |  |  | ＋ |  |  |  | ＋ |  |
| newborn41* |  |  |  |  | ＋ |  |  |  |  |  |  |  |  |  |  |  |  |
| newborn42 | ＋ |  |  |  |  |  |  |  |  |  | ＋ |  |  |  |  |  | ＋ |
| newborn43 |  | ＋ | ＋ |  |  |  |  |  |  | ＋ |  |  | ＋ |  | ＋ |  |  |
| newborn44 |  |  |  |  |  |  |  |  |  |  |  |  |  |  |  |  |  |
| newborn45* | ＋ |  | ＋ |  |  |  |  |  |  |  |  | ＋ |  |  |  |  | ＋ |
| newborn46 |  |  |  |  |  |  |  |  |  |  |  |  |  |  |  |  |  |
| newborn47 |  | ＋ |  |  |  |  |  |  |  |  |  |  | ＋ |  |  |  | ＋ |
| newborn48 | ＋ |  |  |  |  |  |  |  | ＋ |  |  | ＋ |  |  |  |  |  |
| newborn49* |  |  |  |  |  |  | ＋ |  |  |  |  |  |  |  |  |  |  |
| newborn50* |  |  |  |  |  |  |  |  | ＋ | ＋ |  |  |  |  |  | ＋ |  |
| newborn51 | ＋ |  | ＋ |  |  |  |  |  |  |  |  |  |  |  | ＋ |  | ＋ |
| newborn52 |  |  | ＋ |  |  |  |  |  |  | ＋ |  |  |  |  |  |  |  |
| newborn53 |  |  |  |  | ＋ |  |  |  |  |  |  |  |  | ＋ |  |  |  |
| newborn54* | ＋ |  |  |  |  |  |  | ＋ |  |  |  | ＋ |  |  |  |  |  |
| newborn55 |  |  |  |  |  |  |  |  |  |  |  |  |  |  |  |  | ＋ |
| newborn56 | ＋ |  | ＋ |  |  |  |  |  |  |  |  |  |  |  | ＋ |  | ＋ |
| newborn57 |  | ＋ |  |  |  |  |  |  |  |  |  |  | ＋ |  | ＋ |  |  |
| newborn58* | ＋ |  |  | ＋ |  |  |  |  |  |  | ＋ |  |  |  |  |  |  |
| newborn59 |  |  |  |  |  |  |  |  |  |  |  |  |  |  |  |  |  |
| newborn60 |  |  | ＋ |  |  |  |  |  | ＋ |  |  |  |  |  |  | ＋ |  |
| newborn61 | ＋ | ＋ |  |  | ＋ |  |  |  |  |  | ＋ | ＋ |  |  |  |  |  |
| newborn62 |  |  |  |  |  |  |  |  |  |  |  |  |  |  |  |  |  |
| newborn63* | ＋ |  |  | ＋ |  |  |  |  |  | ＋ |  |  |  |  | ＋ |  | ＋ |
| newborn64 |  |  |  |  |  |  |  |  |  |  |  |  |  |  |  |  |  |
| newborn65 | ＋ |  |  |  |  |  |  |  |  |  |  |  |  |  |  |  |  |
| newborn66 |  | ＋ |  |  |  |  |  |  |  |  |  |  | ＋ |  |  |  |  |
| newborn67 |  |  |  |  |  |  |  |  |  |  |  | ＋ |  |  |  |  | ＋ |
| newborn68* | ＋ |  | ＋ |  |  |  |  |  |  |  | ＋ |  |  |  |  |  | ＋ |
| newborn69 |  |  |  | ＋ |  |  |  |  |  |  |  |  |  |  | ＋ | ＋ |  |
| newborn70 |  | ＋ |  |  |  |  |  |  | ＋ |  |  |  |  |  |  |  |  |
| newborn71* | ＋ |  |  |  |  |  |  |  |  | ＋ | ＋ |  |  |  |  |  | ＋ |
| newborn72 |  |  |  |  |  |  |  |  |  | ＋ |  |  |  |  |  |  | ＋ |
| newborn73 | ＋ |  |  |  |  |  |  | ＋ |  |  | ＋ |  |  |  |  |  |  |
| newborn74 |  |  |  |  |  |  |  |  |  |  |  |  |  |  |  |  |  |
| newborn75 | ＋ | ＋ |  |  |  |  |  |  |  |  |  | ＋ |  |  |  |  |  |
| newborn76 |  |  |  |  |  |  |  |  |  |  |  |  |  |  | ＋ |  |  |
| newborn77* |  |  |  |  |  |  |  |  |  | ＋ |  |  |  |  |  |  | ＋ |
| newborn78 |  |  |  |  | ＋ |  |  |  |  |  |  |  | ＋ | ＋ |  |  |  |
| newborn79 |  |  |  |  |  |  |  |  | ＋ |  |  |  |  |  |  | ＋ |  |
| newborn80 | ＋ |  | ＋ |  |  |  |  |  |  | ＋ |  | ＋ |  |  |  |  | ＋ |
| newborn81 |  |  |  |  |  |  |  |  |  |  | ＋ |  |  |  |  |  |  |
| newborn82* | ＋ | ＋ |  |  |  |  |  |  |  |  |  |  |  |  |  |  | ＋ |

*“*” Staphylococcus epidermidis isolated from faeces on the third day is different with that from colostrum about resistance phenotypes and ARGs correspondingly.*

*“+” resistance to antibiotics or positive for ARG*

Table S19 Resistance phenotypes and ARGs of Staphylococcus epidermidis isolated from ward air

| ward air isloates | Ampicillin | Tetracycline | Erythromycin | Cefotaxime | Kanamycin | Vancomycin | Ofloxacin | Chloramphenicol | Sulfamethoxazole | Penicillin | blaTEM | ampc | tetm | aac6ib | ermb | sul2 | mecA |
| --- | --- | --- | --- | --- | --- | --- | --- | --- | --- | --- | --- | --- | --- | --- | --- | --- | --- |
| 1* | ＋ |  | ＋ |  |  |  |  |  | ＋ |  |  |  |  |  |  |  |  |
| 2 | ＋ | ＋ |  |  |  |  |  |  |  |  |  | ＋ | ＋ |  |  |  |  |
| 3* | ＋ | ＋ |  |  | ＋ |  |  |  |  |  |  |  |  |  |  |  | ＋ |
| 4* |  |  |  |  |  |  |  |  |  | ＋ |  |  |  |  |  |  |  |
| 5* |  | ＋ | ＋ |  |  |  |  |  |  |  |  |  |  |  | ＋ |  |  |
| 6* | ＋ |  |  |  | ＋ |  |  |  |  |  |  |  |  |  |  |  | ＋ |
| 7 | ＋ |  |  |  |  |  |  |  |  |  |  |  |  |  |  |  | ＋ |
| 8* |  |  | ＋ |  |  |  |  |  | ＋ |  |  |  |  |  |  |  |  |
| 9* | ＋ |  |  |  |  |  |  | ＋ |  |  |  |  |  |  |  |  | ＋ |
| 10* | ＋ |  |  |  |  |  |  |  | ＋ |  |  |  |  |  |  |  | ＋ |
| 11 |  | ＋ | ＋ |  |  |  |  |  |  | ＋ |  |  | ＋ |  | ＋ |  |  |

*“*” Staphylococcus epidermidis isolated from ward air is different with that from faeces on the third day about resistance phenotypes and ARGs.*

*“+” resistance to antibiotics or positive for ARG*

**3. Reference**

1. Hammerum AM, Jensen LB, Aarestrup FM: **Detection of the satA gene and transferability of virginiamycin resistance in Enterococcus faecium from food-animals**. *FEMS microbiology letters* 1998, **168**(1):145-151.

2. Pu W, Su Y, Li J, Li C, Yang Z, Deng H, Ni C: **High incidence of oxacillin-susceptible mecA-positive Staphylococcus aureus (OS-MRSA) associated with bovine mastitis in China**. *PloS one* 2014, **9**(2):e88134.

3. Huang Y, Zhang L, Tiu L, Wang HH: **Characterization of antibiotic resistance in commensal bacteria from an aquaculture ecosystem**. *Frontiers in microbiology* 2015, **6**:914.

4. Ceuppens S, Delbeke S, De Coninck D, Boussemaere J, Boon N, Uyttendaele M: **Characterization of the Bacterial Community Naturally Present on Commercially Grown Basil Leaves: Evaluation of Sample Preparation Prior to Culture-Independent Techniques**. *International journal of environmental research and public health* 2015, **12**(8):10171-10197.

5. Malhotra-Kumar S, Lammens C, Piessens J, Goossens H: **Multiplex PCR for simultaneous detection of macrolide and tetracycline resistance determinants in streptococci**. *Antimicrobial agents and chemotherapy* 2005, **49**(11):4798-4800.

6. Zhang L, Kinkelaar D, Huang Y, Li Y, Li X, Wang HH: **Acquired antibiotic resistance: are we born with it?***Applied and environmental microbiology* 2011, **77**(20):7134-7141.

7. Park CH, Robicsek A, Jacoby GA, Sahm D, Hooper DC: **Prevalence in the United States of aac(6')-Ib-cr encoding a ciprofloxacin-modifying enzyme**. *Antimicrobial agents and chemotherapy* 2006, **50**(11):3953-3955.

8. Jamal W, Rotimi VO, Albert MJ, Khodakhast F, Udo EE, Poirel L: **Emergence of nosocomial New Delhi metallo-beta-lactamase-1 (NDM-1)-producing Klebsiella pneumoniae in patients admitted to a tertiary care hospital in Kuwait**. *International journal of antimicrobial agents* 2012, **39**(2):183-184.

9. Liu YY, Wang Y, Walsh TR, Yi LX, Zhang R, Spencer J, Doi Y, Tian G, Dong B, Huang X *et al*: **Emergence of plasmid-mediated colistin resistance mechanism MCR-1 in animals and human beings in China: a microbiological and molecular biological study**. *The Lancet Infectious diseases* 2016, **16**(2):161-168.

10. Jiang L, Hu X, Yin D, Zhang H, Yu Z: **Occurrence, distribution and seasonal variation of antibiotics in the Huangpu River, Shanghai, China**. *Chemosphere* 2011, **82**(6):822-828.

11. Excoffier L, Smouse PE, Quattro JM: **Analysis of molecular variance inferred from metric distances among DNA haplotypes: application to human mitochondrial DNA restriction data**. *Genetics* 1992, **131**(2):479-491.

12. Suzuki MT, Taylor LT, DeLong EF: **Quantitative analysis of small-subunit rRNA genes in mixed microbial populations via 5'-nuclease assays**. *Applied and environmental microbiology* 2000, **66**(11):4605-4614.

13. Thomas JC, Vargas MR, Miragaia M, Peacock SJ, Archer GL, Enright MC: **Improved multilocus sequence typing scheme for Staphylococcus epidermidis**. *Journal of clinical microbiology* 2007, **45**(2):616-619.
